# Does the problem begin at the beginning? Medical students’ knowledge and beliefs regarding antibiotics and resistance: a systematic review

**DOI:** 10.1186/s13756-020-00837-z

**Published:** 2020-11-03

**Authors:** Natalia Nogueira-Uzal, Maruxa Zapata-Cachafeiro, Olalla Vázquez-Cancela, Ana López-Durán, Maria T. Herdeiro, Adolfo Figueiras

**Affiliations:** 1 Department of Preventive Medicine and Public Health, University of Santiago de Compostela, 15786 Santiago de Compostela, Spain; 2 Consortium for Biomedical Research in Epidemiology and Public Health (CIBER en Epidemiología Y Salud Pública- CIBERESP), Santiago de Compostela, Spain; 3 Department of Preventive Medicine, Santiago de Compostela University Teaching Hospital, Santiago de Compostela, Spain; 4 Department of Clinical Psychology and Psychobiology, University of Santiago de Compostela, Santiago de Compostela, Spain; 5 Department of Medical Sciences and Institute of Biomedicine, University of Aveiro (iBIMED-UA), Aveiro, Portugal; 6 Health Research Institute of Santiago de Compostela (Instituto de Investigación Sanitaria de Santiago de Compostela - IDIS), Santiago de Compostela, Spain

**Keywords:** Clinical education, Antibiotics, Antimicrobial resistance, Knowledge, Beliefs

## Abstract

**Background:**

Studies have detected that prescribers display gaps in knowledge and inappropriate attitudes regarding antibiotics and resistances, but it is not known whether these are generated during professional practice or derive from the undergraduate stage of their education. Accordingly, the aim of this study was to identify medical students’ knowledge, beliefs and attitudes regarding antibiotic use and antibiotic resistance, and whether these change over the course of their time at medical school.

**Methods:**

We conducted a search of the MEDLINE and EMBASE databases, and included studies that measured knowledge and/or beliefs and/or attitudes regarding antibiotic prescribing and/or resistance, among medical students.

**Results:**

Of the 509 studies retrieved, 22 met the inclusion criteria. While medical students perceived resistance as posing a major public health problem, both worldwide and in their own countries, students in the last two course years were more aware of overprescription of antibiotics in general, and of broad-spectrum antibiotics, at their teaching hospital. There was a considerable lack of knowledge about the treatment of high-incidence infections, and upper respiratory tract infections in particular (41–69% of participants believed antibiotics to be useful for treating these), without any differences by course year. Students were conscious of their personal shortcomings and thus showed willing to improve their education.

**Conclusions:**

Future physicians display important gaps in knowledge, particularly in terms of treatment of high-incidence infections. This finding may be of use when it comes to designing more effective training in antibiotic stewardship for undergraduates.

## Background

Antibiotic resistance is a major public health problem worldwide, which affects developed and developing countries alike, in that it entails important consequences in terms of mortality, morbidity and health costs [1, 2]. Excessive and inappropriate use of antimicrobials is the principal cause of development of resistance [3, 4]. It is estimated that over one third of all antibiotic prescriptions are unnecessary [5].

While many factors influence such misprescription, some of the most important factors are prescribers’ lack of knowledge and favourable attitude to prescribing [6]. It is not known whether this behaviour pattern is generated during professional practice—influenced by extrinsic factors [7] (patients, healthcare system, pharmaceutical industry)—or whether it derives from inadequate training as undergraduates.

The undergraduate stage of medical education is ideal for acquiring knowledge and generating appropriate beliefs and attitudes regarding antibiotics and resistance, since, unlike the postgraduate stage, extrinsic factors have hardly any influence on the generation of knowledge and attitudes [7]. However, we know of no systematic review which could help identify what knowledge, beliefs and attitudes (KBA) medical students have with respect to antibiotics and antibiotic resistance. This could be of great use in terms of providing more effective training in antibiotic stewardship for undergraduates. Accordingly, the main aim of this study was to identify students’ KBA regarding antibiotic use and resistance, and analyse whether these change over the course of their time at medical school. By way of a secondary aim, we proposed to analyse antibiotic self-medication practices reported in the studies reviewed.

## Methods

### Search strategy

For review purposes, we conducted a search of the MEDLINE-PubMED scientific database and EMBASE for all papers published from January 2000 to 22 March 2019. The review itself was performed in accordance with the Preferred Reporting Items for Systematic Reviews and Meta-Analyses (PRISMA) guidelines. The search strategy was designed to identify relevant studies addressing medical students’ KBA regarding antibiotic prescribing and antibiotic resistance, using the following search terms: "medical AND (students OR undergraduate OR faculty) AND (antibiotic OR antimicrobial OR antimicrobial drugs OR antimicrobial resistance OR antibiotic misuse OR antibiotic prescription OR antimicrobial stewardship) AND (knowledge OR attitude OR perceptions OR beliefs) AND (survey OR questionnaire)". No other type of search restriction was applied (language, type of paper or population).

### Study-selection criteria

Studies were deemed eligible for review if they met the following criteria: (i) the target population was required to include medical students, and where the study population included non-medical students or physicians, data were solely extracted from medical students; and, (ii) in terms of outcome measures, studies had to measure knowledge and/or beliefs and/or attitudes regarding antibiotic prescribing and/or antibiotic resistance.

Titles and abstracts were screened by two authors (NN and MZ), working independently. All papers identified as potentially relevant were then reviewed by four of the authors (NN, MZ, MTH and OV), who decided whether or not these met the selection criteria. In case of disagreement, the paper in question was examined by AF, who took the final decision.

### Data-extraction

Data were extracted by two of the authors (NN, MZ). For every study included in the review, the following parameters and characteristics were recorded (see Table 1): author(s); year of publication; country; academic year; sample size; and year and method of data collection. As students' knowledge and beliefs would change over the course of medical school, results have been stratified into two categories: (1) last two course years (according to each country’s study programme); and, (2) the remaining course years, or studies that did not differentiate between course years. Studies conducted on students in their final and/or penultimate course years are shown in the first part of the table, while those referring to the remaining course years are shown in the second part of the table.

**Table 1:** Studies included in the systematic review and results

Authors	Year	Country	Academic year	Sample size (n)	Year Data collected	Data-collection method (questionnaire)	Appraisal tool for Cross-Sectional Studies (AXIS)	Results		
								Knowledge and sources of information		
								Knowledge (% correct responses)^a^	Sources of information/teaching used (%)^a^	Usefulness of the different sources of information^a^ (% students who consider it useful)
*Last courses*										
Sanchez-Fabra et al*. *[32]	2019	Spain	Final Year	441	2015	Online	1, 2, 4, 5, 8, 9, 11, 12, 15, 16, 17, 18, 19	*NE*	FL (59–93) CC (42.2–91.2) NT (65.1) OHSP (67.3) OTHS (71.7)	FL (59.4–68.8) CC (49.2–76.9) NT (40.7) OHSP (62.3) OTHS (62.9)
Rusic et al*. *[31]	2018	Croatia	5th and 6th	115	2017	Paper-based	1, 2, 4, 5, 6, 8, 9, 10, 11, 12, 15, 16 ,17, 18, 19, 20	KAMP (47–92)	TB (91.0) MJ (38.5) NT (62.8–73.1) ABG (69.2)	*NE*
Dutt et al*. *[29]	2018	India	Final year	76	2017	Paper-based	1, 2, 4, 5, 8, 10, 11, 13, 15, 16, 17, 18, 20	KAMP (14.5–73.7)	*NE*	*NE*
Weier et al*.* [27]	2017	Australia	Final year	191	2015	Online	1, 2, 4, 5, 7, 8, 9, 10, 11, 12, 15, 16, 17, 18, 19, 20	*NE*	*NE*	*NE*
Tayyab et al*.* [26]	2017	Pakistan	4th, 5th	223	2016	Paper-based	1, 2, 4, 5, 8, 9, 11, 12, 15, 16, 17, 20	KAMP (57–96)	*NE*	*NE*
Wasserman et al*.* [25]	2017	South Africa	Final year	289	2015	Paper-based	1, 2, 4, 5, 8, 9, 10, 11, 12, 13, 15, 16, 17, 18, 19, 20	KAMP (19–59) CRCV [RTICV (15–78), UTICV (69), OTHCV (43–69)]	*NE*	FL (77–81) CC (54–56) OHSP (85–87) OTHS (63–87)
Chuenchom et al. [23]	2016	Thailand	6th	455	2014	Paper-based	1, 2, 4, 5, 8, 11, 12, 13, 15, 17, 19	KAMP (56.7–58.9) CRCV [RTICV (21.5–84.3), UTICV (51.2), OTHCV (39.1–94.9)]	FL (71.8–79.1) ABG (11.9–52.1) OHSP (10.1–85.0)	*NE*
Yang et al*. *[21]	2016	China	4th	611	2015	Paper-based	1, 2, 4, 5, 8, 9, 10, 11, 12, 13, 15, 16, 17, 18, 19, 20	KAR (11.9–39.1) KAMP (5.0–50.7) CRCV [RTICV (45.0), UTICV (52.3), OTHCV (89.4)]	TB (80.2) MJ (19.7) NT (50.5- 52.1) ABG (8.6–17.3) PHC (13.6) OHSP (22.1–38) OTHS (57.7)	*NE*
Dyar et al. [18]	2013	Scotland, Switzerland, Sweden, Slovenia, Spain, France, England	Final year	338	2012	Online	1, 2, 4, 5, 8, 9, 11, 12, 15, 16, 17, 18, 19	KAR (59–83)	*NE*	*NE*
Dyar et al. [17]	2013	France	5th, 6th	60	2012	Online	1, 2, 4, 8, 9, 11, 12, 15, 16, 17, 18, 19	KAR (22–72)	ABG (62)	*NE*
Thriemer et al. [14]	2013	DR Congo	Final year	106	2011	Paper-based	1, 2, 4, 8, 9, 10, 11, 12, 13, 15, 16, 17, 18, 19, 20	KAR (4.3–42.3) KAMP (61.9–94.3) CRCV [OTHCV (20.7–74.5)]	NT (41.5) ABG (27.3–68.9) PHC (71.7)	*NE*
Abbo et al. [16]	2013	United States	4th	317	2012	Online	1,2,4,5,8,9,10,11,12,13,15,16,17,18,19	KAR (21–57) KAMP (32–91) CRCV [RTICV (87), UTICV (45), OTHCV (59)]	TB (46) MJ (55) NT (41–90) ABG (29–49) PHC (3) OHSP (54–80)	*NE*
Huang et al*. *[13] *(last year)*	2013	China	Last year	241	Unknown	Paper-based	1, 2, 4, 8, 9, 10, 11, 12, 13, 15, 16, 17, 19, 20	KAMP (31.0–93.5)	*NE*	*NE*
Ibia et al*.* [11]	2005	United States	Senior students	989	1999–2000	Paper-based	1, 2, 4, 5, 8, 10, 11, 12, 15, 16, 17, 18	CRCV [RTICV (24.3–65.6), OTHCV (38.6–90.9)]	*NE*	FL (7.1–21.4) CC (15.1) OHSP (15.8–26.1)
*Other courses*										
Hu et al*. *[30]	2018	China	All	1819	2015	Online	1, 2, 4, 5, 6, 10, 11, 12, 15, 16, 17, 18, 19, 20	*NE*	*NE*	*NE*
Harakeh et al. [19]	2015	Saudi Arabia	All	1042	2013	Interview	1, 2, 4, 5, 8, 9, 10, 11, 12, 13, 15, 16, 17, 18, 19, 20	KAMP (48.2–93.7)	NT (41.2) OHSP (43.6) OTHS (47.2)	*NE*
Minen et al. [12]	2010	United States	All	304	Unknown	Online	1, 2, 4, 8, 9, 10, 11, 12, 15, 16, 17, 18, 19	*NE*	TB (53.3) MJ (18.8–38.5) NT (4.3–60.2) ABG (18.4–33.2) PHC (63.5) OHSP (58.9–63.5)	*NE*
Hoque et al*. *[24]	2016	Bangladesh	3th, 4th, 5th	107	2015	Paper-based	1, 2, 3, 4, 5, 6, 7, 8, 9, 10, 11, 12, 14, 15, 16, 17, 18, 19, 20	*NE*	*NE*	*NE*
Haque et al. [22]	2016	Malaysia	3rd, 4th, 5th	142	2015	Paper-based	2, 8, 9, 10, 13, 16, 17, 18, 19, 20	KAR (35–49)	ABG (45)	*NE*
Padmanabha et al*. *[28]	2018	India	2nd	142	2016	Paper-based	1, 2, 5, 4, 11, 15, 16, 17, 18, 20	*NE*	*NE*	*NE*
Sharma et al. [20]	2015	India	2nd	120	2014	Paper-based	1, 2, 4, 8, 9, 10, 11, 12, 13, 15, 16, 17, 19, 20	CRCV (29.2–87.5)	*NE*	*NE*
Khan et al. [15]	2013	India	2th	97	Unknown	Paper-based	1, 2, 4, 8, 9, 10, 11, 12, 13, 15, 16, 17, 18, 19, 20	KAMP (32–77.3)	*NE*	*NE*
Huang et al*. *[13] (1st year)	2013	China	1st year	262	Unknown	Paper-based	1, 2, 4, 8, 9, 10, 11, 12, 13, 15, 16, 17, 19, 20	KAMP (23.1–88.2)	*NE*	*NE*

Authors	Results				Attitudes (%)	Behaviour (%)
	Beliefs					
	Antibiotic resistance as a problem (%)	Overused antibiotics (%)	Contributors to resistance (%)	Others (%)		
*Last courses*						
Sanchez-Fabra et al*. *[32]	*NE*	*NE*	*NE*	PPAB (24.3 -94.8)	ABRE (40.6)	*NE*
Rusic et al*. *[31]	FC (84.6)	OUG (82.1)	INH (47.7) TMBS (83.3) TLD (89.7) IUAB (80.8–88.5) LSU (83.3) PICM (88.5)	PPAB (44.9 74.4) RSP [OPR (48.7–79.5)]	ABRE (74.4–83.3)	*NE*
Dutt et al*. *[29]	*NE*	*NE*	TMP (53.9) IUAB (42.1–78.9) LSU (65.8)	*NE*	*NE*	SSM (76.4) SMRTI (48.7) ICU (28.9–50)
Weier et al*. *[27]	*NE*	*NE*	TLT (75.2) TLD (91.9) IUAB (95.3–96.6)	PPAB (67) CABK (38.8–89.7)	*NE*	*NE*
Tayyab et al*. *[26]	WP (97) NP (84) TH (61)	*NE*	*NE*	*NE*	*NE*	SSM (41) SMRTI (82) ICU (14–78)
Wasserman et al*. *[25]	NP (87) TH (61)	OUN (92) OUTH (63)	TMBS (88) IUAB (98) PHH (62)	PPAB (13–87) RSP [ABDP (13)]	ABRE (90–95)	*NE*
Chuenchom et al. [23]	NP (84.6) TH (75.4)	OUN (98.2) OUTH (94.3)	INH (85.3) TMBS (99.1) PHH (99.6)	PPAB (87.2–99.7) CABK (71.4) RSP [ABDP (99.1)]	ABRE (98.9–99.8)	*NE*
Yang et al*. *[21]	NP (91.6) TH (85.0)	OUN (84.8) OUTH (39.4)	INH (24.6) TMBS (32.5) IUAB (92.3) PICM (70.9)	PPAB (25.6–54.1) RSP [ABDP (76.9)]	ABRE (85.9–90.4)	*NE*
Dyar et al. [18]	NP (92) TH (79) FC (98)	*NE*	TMP (95) TMBS (96) TLT (55) TLD (68) LSU (76) PHH (42) PICM (67)	CABK (40–92) RSP [OPR (66)] RSP [ABDP (60–89)]	ABRE (67–92)	*NE*
Dyar et al. [17]	NP (94) TH (69) FC (96)	*NE*	TMP (98) TMBS (92) TLT (60) TLD (63) LSU (75) PHH (55) PICM (80)	CABK (33–97) RSP [OPR (63)] RSP [ABDP (26)]	ABRE (79)	*NE*
Thriemer et al. [14]	WP (85.5) NP (92.9) FC (67.4)	*NE*	TMP (69.0) TLD (82.1–89.7) IUAB (83.2) PICM (64.1)	CABK (85.7)	*NE*	*NE*
Abbo et al. [16]	NP (98) TH (97)	OUN (94) OUTH (65)	INH (70) TMBS (95) IUAB (97) PICM (83)	PPAB (31–63) RSP [ABDP (20)]	ABRE (79–90)	*NE*
Huang et al*. *[13] *(last year)*	NP (82.6)	OUG (91.7)	TMP (81.4)	*NE*	ABRE (74.5–87.0)	SMRTI (14.5–56.4)
Ibia et al*.* [11]	*NE*	*NE*	*NE*	*NE*	*NE*	*NE*
*Other courses*						
Hu et al*.* [30]	*NE*	*NE*	*NE*	*NE*	*NE*	SSM (27.0) SMRTI (30.7–55.6)
Harakeh et al. [19]	*NE*	*NE*	INH (95.0)	*NE*	*NE*	SSM (49) SMRTI (61.8)
Minen et al. [12]	*NE*	OUG (79.5) OUTH (36.5)	*NE*	*NE*	ABRE (65.8)	*NE*
Hoque et al*.*[24]	NP (99) TH (59)	*NE*	TMP (67.3) TMBS (66.4) TLT (54.2) TLD (54.2) LSU (65.5) PHH (60.7) PICM (81.3)	CABK (64.5–77.5) RSP [OPR (87)] RSP [ABDP (32.7)]	*NE*	*NE*
Haque et al. [22]	NP (83) TH (63) FC (42)	*NE*	TMP (88) TMBS (86) TLT (72) TLD (56) LSU (75) PHH (49) PICM (78)	CABK (53.6–80.3) RSP [ABDP (23)]	ABRE (88)	*NE*
Padmanabha et al*.* [28]	*NE*	*NE*	INH (59.0) TMBS (36.7) TLT (47.5) TLD (38.9) IUAB (35.3–49.6) LSU (48.9) PHH (33.1)	*NE*	*NE*	*NE*
Sharma et al. [20]	*NE*	OUG (99.2)	TMP (96.7) TLT (100) IUAB (100)	CABK (7.5–93.3) RSP [ABDP (72.5)]	ABRE (98)	*NE*
Khan et al. [15]	WP (90.7) NP (88.7) TH (68)	*NE*	TMP (78.3) TMBS (69) TLT (49.5) TLD (58.0) IUAB (56.7) LSU (54.6) PICM (39.0)	RSP [OPR (53.6)]	*NE*	SSM (7.2) SMRTI (56–59.8) ICU (13.4–69)
Huang et al*. *[13] (1st year)	NP (67.2)	OUG (87.2)	TMP (72.9)	*NE*	ABRE (61.1–89.3)	SMRTI (6.4–20.6)

^a^In some studies, this is measured via different items. The response range for the items comprising this construct is shown

NE, not explored. KAR, knowledge about antimicrobial resistance (% students with correct knowledge). KAMP, knowledge about antibiotic use and prescription (% students with correct knowledge). CRCV, correct responses in clinical vignettes (% students with correct knowledge). RTICV, respiratory tract infection clinical vignettes. UTICV, urinary tract infection clinical vignettes. OTHCV, other clinical vignettes. FL, formal lectures. TB, textbooks. MJ, medical journals. CC, clinical cases and clinical rotation. NT, new technologies. ABG, antibiotic guidelines. PHC, pharmaceutical companies. OHSP, other house staff physicians. OTHS, other sources. WP, perception about antibiotic resistance as a worldwide problem. NP, perception of antibiotic resistance as a national problem. TH, perception of antibiotic resistance as a problem at their teaching hospital. FC, perception of antibiotic resistance as a problem in their future career. OUG, overused, generally. OUN, overused, nationally. OUTH, overused at teaching hospitals. INH, inherent in the use of antibiotics. TMP, too many antibiotic prescriptions. TMBS, too many broad-spectrum antibiotics used. TLT, too long treatment. TLD, too low dosage or treatment not completed. IUAB, inappropriate use of antibiotics. LSU, excessive use in livestock. PHH, poor hand hygiene. PICM, poor infection control measures. PPAB, medical students’ perceptions of preparedness in antimicrobial resistance. CABK, confidence in antibiotic knowledge or antibiotic prescription. RSP, responsibility. ABDP, perception of development of new antibiotics. OPR, own professional responsibility. ABRE, attitude to integrating more training in or education about antibiotics and resistance. SSM, students’ self-medication with antibiotics. SMRTI, self-medication with antibiotics for respiratory tract infections. ICU, incorrect use of antibiotics

To extract information on knowledge, beliefs, attitudes and behaviours regarding antibiotics and antibiotic resistance from the studies included, the following process was applied:

1. relevant data in each study were extracted and respectively listed in three tables according to the medical students’ answers (available as Additional Files). Additional File 1 contains information regarding knowledge about antibiotics and resistance, and the sources of information used by students. Additional File 2 covers attitudes and beliefs, and Additional File 3 behaviour with antibiotics;
2. in order to group the information into categories, the issues were evaluated by a panel of experts (clinical pharmacologists, psychologists, public health experts, pharmaco-epidemiologists) with experience in studies on attitudes and knowledge regarding antibiotics [8, 9]. The categories and subcategories established are shown in Table 2; and,
3. the respective studies’ results for each of the categories and subcategories established are shown in Table 1. Where, in any given study, a number of issues belong to a single category and/or subcategory, and yield different results, the range of values of the responses is shown. Table 1 shows a cell with the abbreviation NE (Not explored) to cover any case where a given study might not have taken this category/subcategory into account. This information has also been added as a footnote.

**Table 2:** Classification (and acronyms) of knowledge, attitudes, beliefs and behaviour items

Classification	Acronym
*Knowledge*	
Correct knowledge about antimicrobial resistance	KAR
Knowledge about antimicrobial prescription	KAMP
Correct responses in clinical vignettes, resolution of clinical cases involving antimicrobial prescription including clinical cases related to:	CRCV
Respiratory tract infections	RTICV
Urinary tract infections	UTICV
Other clinical cases	OTHCV
*Sources of information and its usefulness*	
Formal lectures	FL
Textbooks	TB
Medical journals	MJ
Clinical cases and clinical rotation	CC
New technologies such as internet, uptodate, wikipedia, webcasts, podcasts, smartphone applications	NT
Antibiotic guidelines	ABG
Pharmaceutical companies	PHC
Other house staff physicians	OHSP
Other sources	OTHS
*Beliefs*	
Antibiotic resistance as a problem	PARP
Worldwide problem	WP
National problem	NP
Teaching hospital	TH
Their future career	FC
Antibiotics overused	ABOUP
Overused, generally	OUG
Overused, nationally	OUN
Overused at teaching hospitals	OUTH
Contributors to resistance	PCR
Inherent in the use of AB	INH
Too many AB prescriptions	TMP
Too many broad-spectrum AB used	TMBS
Too long treatment	TLT
Too low dosage or treatment not completed	TLD
Inappropriate use of AB	IUAB
Excessive use in livestock	LSU
Poor hand hygiene	PHH
Poor infection control measures	PICM
Preparedness in AB use or AB stewardship	PPAB
Confidence in AB knowledge or AB prescribing	CABK
Responsibility	RSP
Own professional responsibility	OPR
Development of AB	ABDP
*Attitudes*	
Integrating more training or education about antibiotics and resistance	ABRE
*Behaviour*	
Self-medication with AB in general	SSM
Student's self-medication with AB for respiratory tract infections	SMRTI
Incorrect use of AB	ICU

### Quality assessment

To evaluate the quality of the studies selected for inclusion, we used the Appraisal tool for Cross-Sectional Studies (AXIS tool) [10]. Two authors (NN and MZ) independently assessed the quality of the studies included, with any discrepancy or disagreement being resolved through discussion. The above tool consists of a suggested checklist of 20 items with which cross-sectional studies should comply. An important point of this tool is that it evaluates whether a given study’s published conclusions are credible and reliable in light of its designated objective and reported methods, analysis and results.

## Results

### Search results

**Fig. 1:**
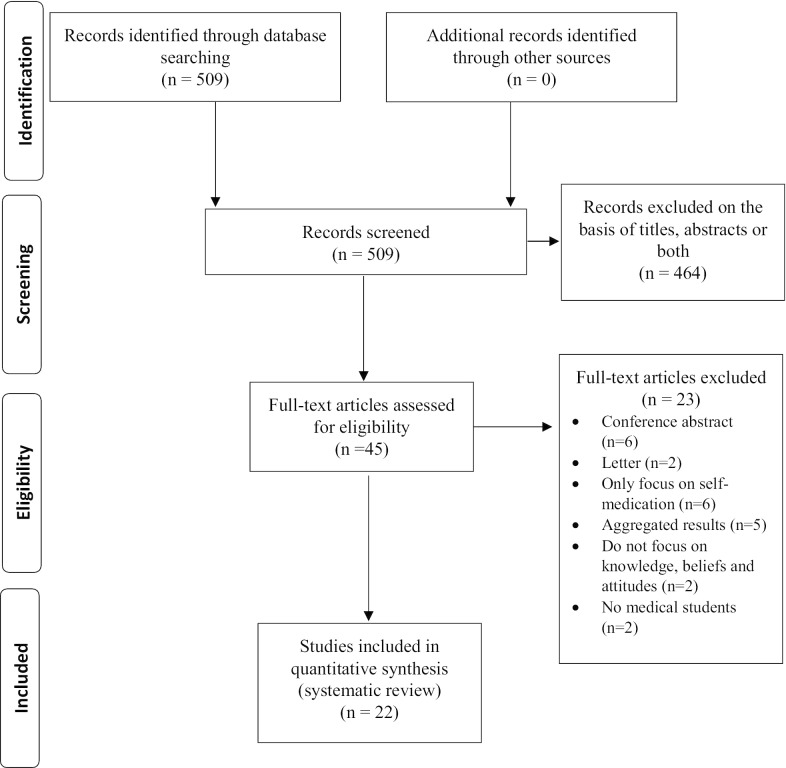
PRISMA Flow chart of study selection

The search strategy identified a total of 509 papers in the MEDLINE PubMed scientific database and EMBASE, which were screened by title and abstract. Of this initial total, 45 papers were subjected to an in-depth reading of the full text, after which 22 were finally included for systematic review purposes (Fig. 1) [11–32].

### Quality assessment

Table 1 lists the AXIS tool items [10] with which each of the papers complied. In general, the studies displayed a similar quality, subject to the main limitations specific to cross-sectional studies, namely, selection bias and small sample size, non-response bias, or the use of questionnaires that had not been previously validated. Even so, we considered that the most important requirements were met by all of the studies, so that 22 papers [11–32] were included in this review.

### Characteristics of selected studies

The general characteristics of the selected studies are summarised in Table 1. The studies were drawn from four different continents, though mainly from Asia (n = 12/22, 55%) [13, 15, 19–24, 26, 28–30]. Four studies were conducted in Europe [17, 18, 31, 32], three in North America [11, 12, 16], two in Africa [14, 25], and one in Australia [27].

The study population ranged from students attending a single institution [12, 14, 15, 17, 20, 22, 24, 28, 29, 31] to those attending various institutions (universities and university hospitals), and from students who were nationals of a single country [11, 13, 16, 19, 21, 23, 25, 27, 30, 32] to those who were nationals of various countries [18].

Study sample size ranged from 60 to 1819 students [17, 30], and response percentages ranged from 6 to 100% [15, 32]. Data-collection methodology also varied among the studies included, with most using online questionnaires [12, 16–18, 27, 30, 32] or paper-based questionnaires [11, 13–15, 20–26, 28, 29, 31], except for one which was interview-based [19].

Of the 22 studies selected, six [14, 15, 20, 22, 24, 32] used a questionnaire which, in its authors’ opinion, was validated (through the undertaking of a pilot study) [22], and seven developed the questionnaire after reviewing the literature and consulting the experts undertaking a pilot study [13, 16–18, 25, 27, 31]. Three studies [12, 19, 21] reported having modified published questionnaires but there was no evidence of the latter’s validation.

Of the 22 studies, 14 made reference to the last two course years [11, 13, 14, 16–18, 21, 23, 25–27, 29, 31, 32]. Three studies pooled all the course years [12, 19, 30] or only made reference to the first year [15, 20, 22, 24, 28]. Huang et al.’s study reported the results for the first and last course years, so that their results are shown as differentiated in both parts of Table 1.

### Knowledge regarding antibiotic use and resistance

Table 1 shows the results with respect to knowledge identified as influencing antibiotic use and resistance, and the sources of information used by medical students, stratified by course year (last two course years vs. initial course years).

Five studies [14, 16–18, 21] on the last two course years and one on the initial course years [22] evaluated whether students were familiar with the principal of ***mechanisms of resistance***, and with the antibiotics and/or microorganisms most frequently associated with resistance. The percentage of correct responses ranged from 4.3 to 83%. With the exception of the paper published by Dyar et al*.* [18], the percentage of correct responses was, in most cases, under 50%.

Nine studies on the last course years [13, 14, 16, 21, 23, 25, 26, 29, 31] and three on the initial course years [13, 15, 19] analysed ***knowledge about antibiotic use*** and prescribing (spectrum of action, use during pregnancy, use in the case of viral or bacterial infections). The items used for evaluation were very diverse (see Additional File 1), as were the results. For instance, in the study by Dutt et al*.* [29], depending on the question posed, the percentages of correct responses might range from 14.5 to 73.7%. This variability in questions and answers makes it impossible to discern differences between students in the last two course years and the remaining medical students.

In studies, in all cases focusing on the final course years, which evaluated the effectiveness of antibiotics in high-incidence infections, e.g., for treating ***colds, influenza and cough ***[13, 23, 31], only 47–60% of students knew that antibiotics were not the treatment of choice. In the study by Huang et al. [13], even though almost 70% of all fifth-year students knew that antibiotics did not cure viral infections, 69% nonetheless thought that the use of antibiotics could ensure a swifter recovery in episodes of colds and coughs [13]. This same study [13] also reported the results obtained among first-year students, among whom only 49.7% responded that antibiotics do not cure viral infections, and only 23.1% knew that they do not help patients recover after influenza or a head cold.

Similarly, there was wide variability both in the ***practical cases*** proposed and in the percentage of cases correctly resolved. As a general rule, however, practical cases of upper respiratory tract infections [11, 16, 21, 23, 25], were observed to display a low percentage of correct responses, with this being less than 50% in a number of cases, as shown in Additional File 1. In this case, no comparison can be made because there were no studies on more junior course years with practical cases of upper respiratory tract infections.

In terms of the **sources of information** used by students in their last two course years, in general, there was little use of antibiotic guidelines or medical journals [14, 16, 17, 21, 23, 31]. This was in sharp contrast to the greater use of new technologies, especially in the most recent studies [31, 32]. When students were asked about the usefulness of different sources of information, the degree of variability among the results was high [11, 25, 32]. The same trend was observed in studies on other course years, such as that by Minen et al. [12].

### Beliefs regarding antibiotic use and resistance

Table 1 shows the results stratified by course year with respect to beliefs identified as influencing antibiotic use and resistance. It will be seen that the majority of students (in the final and initial course years alike) considered antibiotic resistance to be a major ***public health problem***, both worldwide [14, 15, 26] and at a national level [13–18, 21–26]. However, when students were asked whether they saw it as a problem at their own teaching hospital, their belief in it as constituting a problem weakened [15–18, 21–26], regardless of which course year they were in. Similarly, ***overuse of antibiotics*** was perceived as a problem at a general and national level [12, 13, 16, 20, 21, 23, 25, 31] but of less importance at the respondents’ respective teaching hospitals [12, 16, 21, 23, 25]. Here it seems that students in the last two course years [16, 21, 23, 25] are more aware of overprescription at their hospitals (range 39–94%) than are those in the initial course years (a single study [12], with a value of 36%).

Medical students, both in their final [13, 14, 16–18, 21, 23, 25, 27, 29, 31] and initial course years [13, 15, 19, 20, 22, 24, 28], believe that, among the ***factors*** which in greater measure contribute to the appearance of resistance, are excessive prescription of antibiotics [13–15, 17, 18, 20, 22, 24, 29], and inappropriate use [14–16, 20, 21, 25, 27–29, 31] and misuse of broad-spectrum antibiotics [15–18, 21–25, 28, 31] (see Additional File 2). With respect to *broad-spectrum* antibiotics, final-year students showed greater concern than did those in the initial course years (median 92% vs. 69.0%).

When it came to ***personal responsibility***, 48.7–87% of students, whether in their last or initial course years, felt that, as health professionals, physicians were responsible for the problem of resistance [15, 17, 18, 24, 31]. Furthermore, six studies [16–18, 21, 23, 25] on the last two course years evaluated beliefs about the ***development of new antibiotics*** that would solve the problem, with a great disparity of results being in evidence: whereas in three cases [16, 17, 25], less than 30% of students trusted in the development of new antibiotics, in another three studies [18, 21, 23] over 70% of students did so. The same disparity was to be seen in studies focusing on initial course years [20, 22, 24].

Students generally felt that there were important ***gaps in their education*** in terms of their knowledge of antibiotics [16, 21, 23, 25, 27, 31, 32], and thus showed willing to improve their education in this field [13, 16–18, 21, 23, 25, 31, 32]. However, if one were to focus exclusively on data from the last two academic years, in general students considered themselves equipped for clinical practice (especially for identifying signs of infection and making a clinical decision) [17, 18, 23, 27]. Nonetheless, they were less sure when it came to the choice of the appropriate antibiotic, the management of multiresistant microorganisms or the interpretation of results (antibiograms, cultures). The results obtained by studies conducted on first-year students were very similar.

### Attitudes towards receiving more training

Attitudes to integrating more training in or education about antibiotics and resistance were strongly in favour among students in initial course years (61.1–98%) [12, 13, 20, 22], and remained strongly in favour in the final two years (40.6–99.8%) [13, 16–18, 21, 23, 25, 31, 32], as can be seen in Table 1.

### Self-medication practice

With respect to ***self-medication*** with antibiotics, something evaluated in 5 papers [15, 19, 26, 29, 30], observed values ranged from 7.2 to 76.4% (Table 1), with wide differences between the values reported for the initial course years (7.2–49%) [15, 19, 30] and those of 41–76.4% reported for the last two course years [26, 29]. Inappropriate behaviours such as ***not completing the full antibiotic treatment*** or keeping left-over antibiotics were already evident in the initial course years (13.4–69%) [15] and would not seem to have changed in the last two course years (14–78%) [26, 29] (See Additional File 3). In the case of antibiotic use for specifically treating ***upper respiratory tract infections***, up to 82% of students admitted to having used them [26].

## Discussion

To our knowledge, this is the first systematic review of medical students’ KBA regarding antibiotics and antibiotic resistance. The most important finding of this review is that a high percentage of medical students display serious gaps in their knowledge of the diagnosis and treatment of high-incidence infectious diseases in general, and upper respiratory tract infections in particular. The students are aware of their shortcomings, and would like to improve their training. A second key finding is that, except in certain very specific aspects, (concern about overuse of broad-spectrum antibiotics, and the problem of resistance at their hospital), there would not seem to be great differences between initial- and final-year students. A third key finding is the large heterogeneity, not only in terms of the results obtained and the studies and settings, but also in terms of the methodology used (questionnaires administered; items assessed; response percentages).

With antibiotic resistance being recognised as an ***important public-health*** threat worldwide [33] and physicians being seen as crucially responsible for antibiotic misuse, one strength of this study is having detected that, in many settings, physicians display important shortcomings, dating back to the time of their education, in the treatment of highly common infections. We feel that these results are particularly important, since these shortcomings are easily remediable at this early stage of a medical student’s education: it is simply necessary for the educational and/or health authorities (from a global level all the way down to those responsible for individual educational centres) to ensure that their educational priorities are proportional to the impact which antibiotic misuse has on public health around the world.

To account for the factors that influence antibiotic prescribing, the ***modified knowledge, attitudes, practices (KAP) model*** has been proposed, under which prescribing is jointly influenced by internal (KAP) and external factors (pharmaceutical industry, healthcare system or patient pressure), such that external factors could modulate the acquisition of knowledge of one kind or another, depending on the source of information from which such knowledge comes [34]. According to this model, during the undergraduate stage, medical students are free of biased external influences (e.g., patients and pharmaceutical industry) [35], and information stemming from independent sources could thus generate quality knowledge and attitudes, something that would result in better future behaviours. In light of the results of our review, however, it would seem that proper use is not being made of this opportunity.

This review detected important ***gaps*** in medical students’ knowledge, most notably the fact that in some studies 18–30% of students believe that antibiotics are useful for combating viruses [13, 19] or should be used whenever fever is present [15, 29]. Given the high incidence of these types of diseases -especially those of the upper respiratory tract- [36] such gaps may have an extremely high impact on the overprescription of antibiotics worldwide. Furthermore, these gaps in knowledge are maintained in the final two years of medical education and are consistent with the results of recent reviews targeting practising physicians [6, 7, 37–39], which seem to indicate that the shortcomings of postgraduate physicians may well derive from their undergraduate years.

All this suggests the need to improve medical students’ education, particularly in diseases which, though not severely infectious, nonetheless have a high incidence (influenza, colds, sore throat, urinary infections) and a pronounced impact on the misprescription of antibiotics [40].

The results of this review show that, as a source of information, students often employ the tactic of “*asking a staff physician at your hospital*”, before resorting to clinical practice guidelines. In view of the important shortcomings detected in clinical practice with respect to antibiotic use [41], this tendency may lead to antibiotic misprescribing habits being perpetuated over time. In this regard, we feel that it is fundamental for university students to acquire a critical spirit and the necessary competencies for updating themselves on the diagnosis and treatment of infectious diseases. These competencies could be transferred to senior medical staff during the period of their medical residency.

Although students show concern about resistance as ***a public health problem*** at a global level, this concern tends to become less pronounced when asked about the workplace/learning setting closest to them, such as their teaching hospital. This may be very relevant vis-à-vis their approach to medical practice, since it seems to indicate that they perceive it as a remote problem, in which their capacity for action and contribution is reduced, something that could in turn make for a less favourable attitude to responsible antibiotic use. Possibly contributing to this is the fact that the figures on which they rely come from mass media reporting global data rather than from their teachers with reference to the resistance in evidence at their own teaching hospitals.

Lastly, the inappropriate ***practices*** detected with respect to self-medication with antibiotics for upper respiratory tract infections, such as the suspension of antibiotic treatment before completion of the full course, are consistent with the knowledge and attitudes of medical students, and, in some cases, with those of the general population [42]. Furthermore, these self-medication practices appear to increase in the last two course years, a finding that is consistent with the self-treatment culture observed among physicians and medical students [43].

### Discussion of the methods of the studies included

From a methodological standpoint, the majority of the studies were undertaken with students who attended class. This may generate a *selection bias*, since those being studied are presumably the most motivated students who, in all likelihood, also have the best KBA. Even so, we feel that this in no way diminishes the value of the results of our review, since, if important shortcomings were nonetheless found in education, it follows that in reality these would have been greater still if the entire student population had been evaluated.

Most of the studies do not specify whether they used a fully validated questionnaire and what type of validation was used. It would be of great interest to develop a validated questionnaire with transcultural adaptation to different settings, which would ensure comparability of results.

A further limitation is that there are studies targeting different academic years, which means that they are not altogether comparable. Furthermore, age of access to university may vary among countries, and the fact that students are younger in some Asian countries may be reflected in their answers. Accordingly, in future studies it would be advisable for these questionnaires to be administered in the final academic year of the degree course, or for the results to be broken down by academic year.

### Limitations of systematic review

The principal limitation of our study consists of the difficulty of allocating the items shown in the respective studies’ results to knowledge or attitudes (in some cases without having access to the questionnaire) previously described in earlier studies on practising physicians. Our allocation of the items to knowledge and attitudes may possibly not be in line with that of other authors but we nevertheless feel that this would not alter the main conclusions of this review.

Special mention should also be made of the heterogeneity of results observed among the various studies. This could be due to different factors, such as: (1) different educational systems with different training priorities as regards the treatment of infections and correct use of antibiotics; (2) cultural and educational variations in students’ populations of origin; and, (3) conceivably to a lesser extent, methodological differences in the way of measuring knowledge and attitudes (questionnaires) or biases (participation). These variations between settings in terms of antibiotics have already been described in antibiotic use [44].

Lastly, the results may not be representative of medical students worldwide, since only 22 papers fulfilled the inclusion criteria and most of these were Asian in origin.

## Conclusions

Physicians play a key role in the fight to reduce antibiotic resistance worldwide. Although there is a need for further studies covering more countries and involving a larger number of students, our results would nonetheless seem to suggest that there are important shortcomings in undergraduate education in this field, in many places around the world. It seems necessary to establish or modify educational plans in Medical Faculties to improve education on antibiotics, resistance and treatment of infections (principally those that are least severe and most common). This would result in more prudent prescription of antibiotics, which would doubtless contribute to the control of resistance in the not too distant future.

## Supplementary information

**Additional file 1.** Knowledge items and sources of information.

**Additional file 2.** Attitudes and beliefs items.

**Additional file 3.** Antibiotic behaviour among medical students.

## Data Availability

Not applicable.

## Abbreviations

KBA: Knowledge, beliefs and attitudes
AXIS: Appraisal tool for Cross-Sectional Studies
KAP: Knowledge, attitudes and practices
