# Supplementary material for: Does the problem begin at the beginning? Medical students’ knowledge and beliefs regarding antibiotics and resistance: a systematic review
Source: Antimicrob Resist Infect Control. 2020 Nov 3;9:172. doi: 10.1186/s13756-020-00837-z (PMC7607835; doi:10.1186/s13756-020-00837-z)
Supplement: Supplementary file 1 — Additional file 1. Knowledge items and sources of information. [file 13756_2020_837_MOESM1_ESM.docx]

**Additional File 1.** Knowledge items and sources of information

|  | Sanchez-Fabra *et al*. | Rusic *et a*l. | Hu *et al*. | Dutt *et al*. | Padmanabha *et al*. | Weier *et al*. | Tayyab *et al*. | Wasserman *et al*. | Hoque *et al*. | Chuenchom *et al*. | Haque *et al*. | Yang *et al*. | Sharma *et al*. | Harakeh *et al.* | Dyar *et al*. (Europe) | Dyar *et al.* (France) | Abbo *et al*. | Khan *et al*. | Thriemer *et al*. | Huang *et al*. | Minen *et al*. | Ibia *et al.* |
| --- | --- | --- | --- | --- | --- | --- | --- | --- | --- | --- | --- | --- | --- | --- | --- | --- | --- | --- | --- | --- | --- | --- |
|  | Sample size (n) | | | | | | | | | | | | | | | | | | | | | |
|  | 441 | 78 | 1819 | 76 | 139 | 163 | 223 | 289 | 107 | 455 | 142 | 611 | 120 | 1042 | 338 | 60 | 317 | 97 | 106 | 1236 | 304 | 989 |
| **KAR,** knowledge about antimicrobial resistance | | | | | | | | | | | | | | | | | | | | | | |
| - Respondents stated that methicillin-resistant *Staphylococcus aureus* was <1% 10 years ago  - Respondents stated that methicillin-resistant *Staphylococcus aureus* was responsible for a greater proportion (21%-40%) of *S. aureus* bacteraemia in Malaysia  - Respondents thought that over 1%-20% of *S. aureus* bacteraemias were caused by vancomycin-resistant bacteria in Malaysia  - Respondents believed that over 21%-40% of all bacterial infections in Malaysia (excluding tuberculosis (TB)) were resistant to all known antibiotics |  |  |  |  |  |  |  |  |  |  | 35% |  |  |  |  |  |  |  |  |  |  |  |
|  |  |  |  |  |  |  |  |  |  |  | 42% |  |  |  |  |  |  |  |  |  |  |  |
|  |  |  |  |  |  |  |  |  |  |  | 49% |  |  |  |  |  |  |  |  |  |  |  |
|  |  |  |  |  |  |  |  |  |  |  | 40% |  |  |  |  |  |  |  |  |  |  |  |
| Match the antimicrobial/organism with most likely mechanism of resistance  *-E. coli*/β-lactam resistance  *-S. aureus*/methicillin resistance  *-S. aureus*/ vancomycin intermediate  -Enterococcus/cephalosporin |  |  |  |  |  |  |  |  |  |  |  |  |  |  |  |  |  |  |  |  |  |  |
|  |  |  |  |  |  |  |  |  |  |  |  | 39.1% |  |  |  |  |  |  |  |  |  |  |
|  |  |  |  |  |  |  |  |  |  |  |  | 37.0% |  |  |  |  |  |  |  |  |  |  |
|  |  |  |  |  |  |  |  |  |  |  |  | 27.5% |  |  |  |  |  |  |  |  |  |  |
|  |  |  |  |  |  |  |  |  |  |  |  | 11.9% |  |  |  |  |  |  |  |  |  |  |
| -Methicillin-resistant *Staphylococcus aureus* was responsible for a greater proportion of *A. aureus* bacteraemias in our country today than 10 years ago  -Over 1% of *S. aureus* bacteraemias were caused by vancomycin-resistant bacteria in our country  -Over than 1% of all bacterial infections in Europe (excluding TB, are resistant to all known antibiotics). |  |  |  |  |  |  |  |  |  |  |  |  |  |  | 83%  65%  59% |  |  |  |  |  |  |  |
| -Students knew that methicillin-resistant *Staphylococcus aureus* was responsible for a greater proportion of *A. aureus* bacteraemias in our country today than 10 years ago  -Students felt that vancomycin resistant *S. aureus* were responsible for over 1% of all *S. aureus* blood stream infections in France  -Students believed that over 1% of all bacterial infections in Europe (excluding TB, are resistant to all known antibiotics) |  |  |  |  |  |  |  |  |  |  |  |  |  |  |  | 22%  72%  72% |  |  |  |  |  |  |
| Match the antimicrobial/organism with the most likely mechanism of resistance (efflux pumps, alteration of binding site, thickening of the cell wall, enzymatic or intrinsic/not acquired)  -*E. co*li/ β-lactam resistance  -*S. aureus*/methicillin intermediate  -*S. aureus*/ vancomycin intermediate  -*Enterococcus*/cephalosporin |  |  |  |  |  |  |  |  |  |  |  |  |  |  |  |  | 52%  57%  21%  23% |  |  |  |  |  |
| -Methicillin resistant-*Staphylococcus aureus* is susceptible to: none of those antibiotics.  -In DR Congo, what, according to your information, is the estimated resistance rate of *Klebsiella* to ceftriaxone? (50-75%)  -In DR Congo, what, according to your information, is the estimated resistance rate of *Salmonella Typhi* to cotrimoxazole (Bactrim)? (50-75%) |  |  |  |  |  |  |  |  |  |  |  |  |  |  |  |  |  |  | 30.7%  4.3%  42.3% |  |  |  |
| **KAMP**, knowledge about antimicrobial use and antibiotic prescription | | | | | | | | | | | | | | | | | | | | | | |
| Common cold is a self-limiting disease not requiring antibiotics (yes)  Antibiotics are effective for treating bacterial infections (yes)  Antibiotics could reduce the symptoms of the common cold (don’t agree) |  | >60%  92%  47% |  |  |  |  |  |  |  |  |  |  |  |  |  |  |  |  |  |  |  |  |
| Broad-spectrum antibiotics are better than narrow-spectrum ones (disagree)  Antibiotic usage disturbs the gut flora and causes diarrhoea (agree)  Do you think that antibiotics should be used in any case, once you have fever? (no) |  |  |  | 14.5%  73.7%  23.6% |  |  |  |  |  |  |  |  |  |  |  |  |  |  |  |  |  |  |
| Assessment of respondent’s knowledge regarding antibiotic use  **-**Indiscriminate and injudicious use of antibiotics can lead to ineffective treatment (true)  -Indiscriminate and injudicious use of antibiotics can lead to prolongation of illness (true)  -Indiscriminate and injudicious use of antibiotics can lead to emergence of bacteria resistance (true)  -Indiscriminate and injudicious use of antibiotics can lead to additional burden of medical costs to the patient (true)  -If taken too often, antibiotics are less likely to work in future (true)  -Bacteria are germs that cause common ‘flu and cold (false) |  |  |  |  |  |  | 89%  77%  96%  92%  89%  57% |  |  |  |  |  |  |  |  |  |  |  |  |  |  |  |
| Recognising the spectrum of activity of selected antimicrobial agents  Interpretation of antibiogram and de-escalation  Extended-spectrum beta-lactamase-positive *Escherichia coli* bacteraemia: antimicrobial selection |  |  |  |  |  |  |  | 23%  59%  19% |  |  |  |  |  |  |  |  |  |  |  |  |  |  |
| We should prescribe antimicrobial agents to patients with symptoms of fever, cough, sore throat and runny nose (negative)  We should prescribe antimicrobial agents to patients with diarrhoea and vomiting from food poisoning (negative) |  |  |  |  |  |  |  |  |  | 58.9%  56.7% |  |  |  |  |  |  |  |  |  |  |  |  |
| Recognise the possible risks associated with unnecessary use of antimicrobials  Identify scenarios with potential for unnecessary use of antimicrobials  Recognise the spectrum of activity of selected antimicrobial agents  Extended-spectrum *β-lactamase*-positive *E. coli* bacteraemia: antimicrobial selection |  |  |  |  |  |  |  |  |  |  |  | 50.7%  5.0%  22.3%  5.0% |  |  |  |  |  |  |  |  |  |  |
| Students knew that codeine was or was not an antibiotic  Students knew that antibiotics should be used in the treatment of bacterial infections  Antibiotics should not be used in the treatment of viral infections  Antibiotics are effective in treating bacterial but no viral infections  Students correctly identified penicillin as an antibiotic |  |  |  |  |  |  |  |  |  |  |  |  |  | 48.2%  93.7%  81.9%  75.0%  86.6% |  |  |  |  |  |  |  |  |
| Recognise the possible risks associated with unnecessary use of antimicrobials  Identify scenarios with potential for unnecessary use of antimicrobials  Recognise the spectrum of activity of selected antimicrobial agents  Extended-spectrum *β-lactamase* positive *E. coli* bacteraemia: antimicrobial selection |  |  |  |  |  |  |  |  |  |  |  |  |  |  |  |  | 91%  59%  52%  32% |  |  |  |  |  |
| Respondents were aware that bacteria were not responsible for causing colds and ‘flu.  When I get fever, antibiotics help me to get better more quickly (disagree)  When I have a cold, I should take antibiotics to prevent getting a more serious illness (disagree) |  |  |  |  |  |  |  |  |  |  |  |  |  |  |  |  |  | 77.3%  32%  56% |  |  |  |  |
| Which one of the following antibiotics is safe during pregnancy? amoxicillin  Which one of the following antibiotics has the best activity against anaerobes? metronidazole  Aminoglycosides such as gentamicin are very active if they are administered as follows: parenteral once daily  Which one of the following antibiotics most effectively crosses the blood-brain barrier? ceftriaxone |  |  |  |  |  |  |  |  |  |  |  |  |  |  |  |  |  |  | 89.5%  94.3%  61.9%  71.1% |  |  |  |
| Do you think the use of antibiotics will speed up the recovery of cold, cough? (no)  Can antibiotics cure bacterial infections? (yes)  Can antibiotics cure viral infections? (no) |  |  |  |  |  |  |  |  |  |  |  |  |  |  |  |  |  |  |  | 31.0% (5^th^ y)  23.1%  (1^st^ y)  93.5%  (5^th^ y)  88.2%  (1^st^ y)  69.1%  (5^th^ y)  49.7%  (1^st^ y) |  |  |
| **CRCV,** correct responses in clinical vignettes  **RTICV**, respiratory tract infection clinical vignettes. **UTICV**, urinary tract infection clinical vignettes. **OTHCV**, other clinical vignettes | | | | | | | | | | | | | | | | | | | | | | |
| Summary of knowledge vignettes with the corresponding proportion of correct responses by medical school. Competency assessed:  -Management of uncomplicated urinary tract infection. **UTICV**  -Assessing severity and management of community-acquired pneumonia. **RTICV**  -Management of upper-respiratory tract infection. **RTICV**  -Management of drip-site infection. **RTICV**  -Assessing severity and management of *C. difficile* infection. **OTHCV**  -Peri-operative antibiotic prophylaxis: duration and risks of excessive use. **OTHCV**  -Management of poor response to empirical antibiotic therapy. **OTHCV** |  |  |  |  |  |  |  | 69%  78%  70%  15%  43%  57%  69% |  |  |  |  |  |  |  |  |  |  |  |  |  |  |
| A 20-year-old female, with no underlying disease. Health check-up showed WBC 20-30 cells/HPS and bacteria in urine. No antimicrobial was prescribed. (correct answer: appropriate) **UTICV** |  |  |  |  |  |  |  |  |  | 51.2% |  |  |  |  |  |  |  |  |  |  |  |  |
| A 40-year-old female with a simple cyst on the left breast. Seven days after excision, wound dehiscence occurred. Doctor ordered mupirocin ointment for wound dressing. (correct answer: inappropriate) **OTHCV** |  |  |  |  |  |  |  |  |  | 39.1% |  |  |  |  |  |  |  |  |  |  |  |  |
| A 26-year-old male with allergic rhinitis. He had high-grade fever, myalgia, rhinorrhoea, and sore throat. He was diagnosed with influenza infection. The doctor prescribed amoxicillin 1,500 mg a day for 7 days. (Correct answer: inappropriate) **RTICV** |  |  |  |  |  |  |  |  |  | 84.3% |  |  |  |  |  |  |  |  |  |  |  |  |
| A 25-year-old female, normal labour with grade 2 episiotomy wound. The doctor prescribed amoxicillin 1,500 mg a day for 5 days. (Correct answer: inappropriate) **OTHCV** |  |  |  |  |  |  |  |  |  | 47.0% |  |  |  |  |  |  |  |  |  |  |  |  |
| A 2-year-old boy with watery diarrhoea, no mucous/bloody stool, no fever, no vomiting. Doctor prescribed anti-emetic drug and mineral powder, and suggested a follow-up visit if symptoms worsened. (Correct answer: appropriate) **OTHCV** |  |  |  |  |  |  |  |  |  | 94.9% |  |  |  |  |  |  |  |  |  |  |  |  |
| A 70-year-old male admitted with acute stroke. Five days later he developed left lower lung infiltration with high fever. Doctor ordered ceftriaxone and clindamycin for treatment of aspiration pneumonia. (Correct answer: inappropriate) **RTICV** |  |  |  |  |  |  |  |  |  | 21.5% |  |  |  |  |  |  |  |  |  |  |  |  |
| Competency assessed in clinical vignette:  Diagnosis of community-acquired pneumonia: selection of appropriate antimicrobial and switch intravenous to oral therapy **RTICV**  Recognise *Clostridium difficile* infection secondary to the use of antimicrobials. **OTHCV**  Complicated UTI: appropriate antimicrobial selection and duration of treatment. **UTICV** |  |  |  |  |  |  |  |  |  |  |  | 45.0%  89.4%  52.3% |  |  |  |  |  |  |  |  |  |  |
| Would you like to prescribe antimicrobials in the following situations? (correct response):  -Clinical case 1.  -Clinical case 2.  -Clinical case 5.  -Clinical case 6. |  |  |  |  |  |  |  |  |  |  |  |  | 87.5%  75.0%  29.2%  83.3% |  |  |  |  |  |  |  |  |  |
| Competency assessed in clinical vignette. Diagnosis of community-acquired pneumonia: selection of appropriate antimicrobial and switch intravenous to oral therapy **RTICV**  Competency assessed in clinical vignette. Recognise *Clostridium difficile* infection secondary to the use of antimicrobials **OTHCV** |  |  |  |  |  |  |  |  |  |  |  |  |  |  |  |  | 87%  59% |  |  |  |  |  |
| Competency assessed in clinical vignette. complicated UTI: appropriate antimicrobial selection and duration of treatment **UTICV** |  |  |  |  |  |  |  |  |  |  |  |  |  |  |  |  | 45% |  |  |  |  |  |
| Case vignettes:  -Antibiotic prescription for an 18-month-old, previously healthy child with a 4-day history of yellow-green rhinorrhoea and a bedtime cough who has a temperature of 38.3ºC but is otherwise well. (Best option is c: withhold antibiotic until at least 10 days of cough and nasal mucopurulent drainage without improvement) **RTICV**  -The same child was noted to tug at her right ear. On otoscopic examination, her right eardrum is red. There is no TM bulging. Mobility is ok. Would you prescribe antibiotics today? (Best option is b: no) **OTHCV**  -An 18-month-old is tugging at the right ear. She has manifested crankiness since the previous night. Otoscopic examination reveals an immobile, bulging, dull-yellow right tympanic membrane. Would you prescribe antibiotics today? (Best option is a: yes) **OTHCV**  -An 18-month-old not seen previously is noted to have a dulled right TM: which is in neutral position (not bulging or retracted). It is immobile to pneumo-otoscopy. The left TM is normal. You diagnose otitis media with effusion (i.e., OME, middle ear effusion or secretory otitis media). Would you prescribe an antibiotic at this time? (Best option is b: no) **OTHCV**  -An 18-month-old has temperature of 38.3ºC, mucopurulent nasal discharge of 4 days’ duration intermittent cough. RR=30, and normal otoscopic examination. Sleeping and drinking are reduced. Auscultation reveals bilateral expiratory wheezes and coarse rhonchi in both lung fields. No crackles are hard. Your clinical diagnosis is bronchitis and an URI. This is the child’s first such episode. Assume that you have detected localised, fine crackles at the right base and right axilla. Would you now prescribe antibiotics for possible bronchopneumonia? (Best option is d: I would send for x-ray and treat only if is bronchopneumonia) **RTICV**  -The mother of a 4-year-old (temperature 38.3ºC), states that her child has a sore throat. Physical examination is normal except for pharyngeal erythema and shotty nontender cervical nodes. No tonsillar exudate is noted. No rapid strep test is available because the child’s managed care organisation is cutting costs. At this point you would: (best option is a: ask the lab to process a throat culture. No penicillin/amoxicillin for now. If the culture is positive, prescribe penicillin/amoxicillin by telephone) **RTICV**  -An 18-month-old (who is afebrile) has profuse purulent rhinorrhoea. This child attends day care. After how many days of continuous purulent rhinorrhoea would you prescribe antibiotics? (Best option is d: >10 days) **RTICV** |  |  |  |  |  |  |  |  |  |  |  |  |  |  |  |  |  |  |  |  |  | 63.5%  47.1%  90.9%  38.6%  37%  65.6%  24.3% |
| **SOI** sources of information/teaching used by medical students  **FL** formal lectures, **NT** new technologies, **CC** clinical cases and clinical rotation, **TB** textbooks, **MJ** medical journals, **ABG** antibiotic guidelines, **PHC** pharmaceutical companies**, OHSP** other house staff physicians, **OTHS** other sources. | | | | | | | | | | | | | | | | | | | | | | |
| Which of the following methods have been used at your medical school for teaching about prudent antibiotic use?  -Lectures (with > 15 people) **FL**  -Small group teaching (with <15 people) **FL**  -Discussions of clinical cases and vignettes **CC**  -Active learning assignments (e.g., article  reading, group work, preparing an oral  presentation) **OTHS**  -E‐learning **NT**  -Role play or communication skills sessions dealing with patients demanding antibiotic therapy **CC**  -Infectious diseases clinical placement (i.e., clinical rotation or training in infectious diseases, involving patients) **CC**  -Microbiology clinical placement **CC**  -Peer or near‐peer teaching (i.e., teaching led by other students, or recently qualified doctors) **OHSP** | 93%  59%  91.2%  71.7%  65.1%  42.2%  79.4%  76%  67.3% |  |  |  |  |  |  |  |  |  |  |  |  |  |  |  |  |  |  |  |  |  |
| Students who stated they often/sometimes use the following sources to learn about antimicrobial use and resistance  -textbooks or study guides **TB**  -smartphone apps **NT**  -Wikipedia **NT**  -official guidelines by professional organisations **ABG**  -medical journals **MJ** |  | 91.0%  73.1%  62.8%  69.2%  38.5% |  |  |  |  |  |  |  |  |  |  |  |  |  |  |  |  |  |  |  |  |
| To learn about appropriate antimicrobial use, the best modality is:  -bedside teaching with medical staff **OHSP**  -grand rounds **FL**  -lectures **FL**  -students preferred the international antimicrobial handbook **ABG**  -students preferred the Thai antimicrobial handbook **ABG**  -participants with questions regarding antimicrobial use preferred asking the ward resident **OHSP**  -followed by their attending staff **OHSP**  -and the infectious disease specialist **OHSP** |  |  |  |  |  |  |  |  |  | 85.0%  79.1%  71.8%  52.1%  11.9%  62.6%  57.4%  10.1% |  |  |  |  |  |  |  |  |  |  |  |  |
| Students personally used or consulted antibiotic guidelines when considering an antibiotic for a patient **ABG** |  |  |  |  |  |  |  |  |  |  | 45% |  |  |  |  |  |  |  |  |  |  |  |
| Resources used by medical students to learn about antimicrobial use and resistance, percentage reporting resource used sometimes/often  -textbooks or study guides **TB**  -peers (other students) **OTHS**  -Wikipedia **NT**  -iPhone/smartphone applications **NT**  -non-ID physicians **OHSP**  -infectious disease specialist **OHSP**  -hospital pharmacists **OHSP**  -medical journals **MJ**  -other guidelines by professional organisations **ABG**  -pharmaceutical representatives **PHC**  -Johns Hopkins Antibiotic Guide **ABG**  -Infectious Diseases Society of America guidelines **ABG**  -Sanford guide **ABG** |  |  |  |  |  |  |  |  |  |  |  | 80.2%  57.7%  52.1%  50.5%  38.0%  27.0%  22.1%  19.7%  17.3%  13.6%  11.5%  10.6%  8.6% |  |  |  |  |  |  |  |  |  |  |
| In the past year, respondents had obtained some information on antibiotics from one source or another (87.1%). Among the top three sources of information cited were:  -doctors **OHSP**  -internet **NT**  -"other sources": pharmacists, drugstores, leaflets/publications related to a disease or a health condition, medicine notice/leaflet and the media (TV, newspaper or magazines) **OTHS** |  |  |  |  |  |  |  |  |  |  |  |  |  | 43.6%  41.2%  47.2% |  |  |  |  |  |  |  |  |
| Students had personally used antibiotic guidelines when considering an appropriate antibiotic therapy for a patient. **ABG** |  |  |  |  |  |  |  |  |  |  |  |  |  |  |  | 62% |  |  |  |  |  |  |
| Resources used for learning about antimicrobial prescribing and resistance (% who often or sometimes use source)  -UpToDate **NT**  -iPhone or smartphone application **NT**  -hospital pharmacists **OHSP**  -non-infectious disease physicians **OHSP**  -infectious disease specialists **OHSP**  -medical journals **MJ**  -peers (other students) **OHSP**  -Sanford guide **ABG**  -Infectious Diseases Society of America guidelines. **ABG**  -other guidelines by professional organisations **ABG**  -textbooks or study guides **TB**  -Wikipedia **NT**  -Pharmaceutical representatives **PHC** |  |  |  |  |  |  |  |  |  |  |  |  |  |  |  |  | 90%  83%  80%  80%  72%  55%  54%  49%  29%  48%  46%  41%  3% |  |  |  |  |  |
| Sources of information used about antibiotics and antibiotic prescribing:  antibiotic guidelines **ABG**  WHO guidelines **ABG**  pharmaceutical companies **PHC**  internet **NT** |  |  |  |  |  |  |  |  |  |  |  |  |  |  |  |  |  |  | 68.9%  27.3%  71.7%  41.5% |  |  |  |
| Please indicate how often you use the sources below to inform you about appropriate antimicrobial treatment (all of the time/most of the time/some of the time)  -infectious disease attendings and fellow **OHSP**  -non-infectious disease attendings and fellow **OHSP**  -other house staff physicians **OHSP**  -education from a pharmaceutical representative **PHC**  -Sanford guide **ABG**  -other pocket guide **ABG**  -palm or other handheld application **NT**  -internet **NT**  -medical journals **MJ**  -the medical letter **MJ**  -Webcasts **NT**  -audio podcasts **NT**  -textbooks **TB** |  |  |  |  |  |  |  |  |  |  |  |  |  |  |  |  |  |  |  |  | 58.9%  61.8%  63.5%  63.5%  33.2%  18.4%  51.3%  60.2%  38.5%  18.8%  4.93%  4.3%  53.3% |  |
| **USOI** Usefulness of the different sources of information on antibiotics  **FL** formal lectures, **NT** new technologies, **CC** clinical cases, **TB** textbooks, **MJ** medical journals, **ABG** antibiotic guidelines, **PHC** pharmaceutical companies**, OHSP** other house staff physicians, **OTHS** other sources | | | | | | | | | | | | | | | | | | | | | | |
| How useful were the following methods used at your medical school for teaching about prudent antibiotic use?:  -lectures (with >15 people) **FL**  -small group teaching (with <15 people) **FL**  -discussions of clinical cases and vignettes **CC**  -active learning assignments (e.g. article reading, group work, preparing an oral presentation) **OTHS**  -e‐learning **NT**  -role play or communication skills sessions dealing with patients demanding antibiotic therapy **CC**  -infectious diseases clinical placement (i.e., clinical rotation or training in infectious diseases, involving patients) **CC**  -microbiology clinical placement **CC**  -Peer or near‐peer teaching (i.e., teaching led by other students, or recently qualified doctors) **OHSP** | 59.4%  68.8%  76.9%  62.9%  40.7%  52.1%  76%  49.2%  62.3% |  |  |  |  |  |  |  |  |  |  |  |  |  |  |  |  |  |  |  |  |  |
| Usefulness prescribing and resistance.  Antimicrobial prescribing and resistance. Type of education (Wasserman):  -formal lectures. **FL**  -bedside tutorials. **OHSP**  -consultant rounds. **OTHS**  -registrar rounds. **OTHS**  -problem-based learning **CC**  Antibiotic classes and spectrums of activity.  -formal lectures. **FL**  -bedside tutorials. **OHSP**  -consultant rounds. **OTHS**  -registrar rounds. **OTHS**  -problem-based learning **CC** |  |  |  |  |  |  |  | 77%  85%  87%  66%  54%  81%  87%  75%  63%  56% |  |  |  |  |  |  |  |  |  |  |  |  |  |  |
| The five single most helpful resources for learning about judicious antibiotic prescription and antibiotic resistance in ranking order were:  -interactive sessions between students, residents, and faculty. **OHSP**  -lecture series for medical students. **FL**  -feedback from faculty on diagnosis and antibiotic use in students’ outpatient continuity clinic. **OHSP**  -patient-oriented problem-solving modules completed by small groups of medical students or residents. **CC**  -Grand round presentations. **FL** |  |  |  |  |  |  |  |  |  |  |  |  |  |  |  |  |  |  |  |  |  | 26.1%  21.4%  15.8%  15.1%  7.1% |
