# Supplementary material for: Does the problem begin at the beginning? Medical students’ knowledge and beliefs regarding antibiotics and resistance: a systematic review
Source: Antimicrob Resist Infect Control. 2020 Nov 3;9:172. doi: 10.1186/s13756-020-00837-z (PMC7607835; doi:10.1186/s13756-020-00837-z)
Supplement: Supplementary file 2 — Additional file 2. Attitudes and beliefs items. [file 13756_2020_837_MOESM2_ESM.docx]

| **Additional File 2:** Attitudes and beliefs items | | | | | | | | | | | | | | | | | | | | | | | | | | | | | | | | | | | | | | | |
| --- | --- | --- | --- | --- | --- | --- | --- | --- | --- | --- | --- | --- | --- | --- | --- | --- | --- | --- | --- | --- | --- | --- | --- | --- | --- | --- | --- | --- | --- | --- | --- | --- | --- | --- | --- | --- | --- | --- | --- |
|  | Sanchez-Fabra *et al.* | Rusic *et al.* | Hu *et al.* | Dutt *et al.* | Padmanabha *et al.* | Weier *et al.* | | Tayyab *et al.* | | W asserman *et al.* | | Hoque *et al.* | | Chuenchom *et al.* | | Haque *et al.* | | Yang *et al.* | | Sharma *et al.* | | Harakeh *et al.* | | Dyar *et al*.(Europe) | | Dyar *et al* .(France) | | Abbo *et al.* | | Khan *et al*. | | Thriemer *et al*. | | Huang *et al*. | | Minen *et al.* | | Ibia *et al.* | |
|  | Sample size (n) | | | | | | | | | | | | | | | | | | | | | | | | | | | | | | | | | | | | | |  |
|  | 441 | 78 | 1819 | 76 | 139 | 191 | 223 | | 289 | | 107 | | 455 | | 142 | | 611 | | 120 | | 1042 | | 338 | | 60 | | 317 | | 97 | | 106 | | 1236 | | 304 | | 989 | |  |
| **PARP**, perception about antibiotic resistance like a problem. | | | | | | | | | | | | | | | | | | | | | | | | | | | | | | | | | | | | | | |  |
| **WP**, *perception of antibiotic resistance (ABR) as a worldwide problem:*  ABR is a serious health issue facing the world (somewhat agree/strongly agree) (Tayyab *et al*.)  ABR is an important and serious public health issue facing the world  (agree) (Khan *et al*.)  ABR is a worldwide problem (agree/strongly agree) (Thriemer *et al*.) |  |  |  |  |  |  | 97% | |  | |  | |  | |  | |  | |  | |  | |  | |  | |  | | 90.7% | | 85.5% | |  | |  | |  | |  |
| **NP,** *perception of ABR as a national problem:*  ABR is a serious health issue in our country (somewhat agree/strongly agree) (Tayyab *et al*.)  ABR is a significant problem in South Africa (agree/strongly agree) (Wasserman *et al*.)  Students thought that ABR was a national problem (Hoque *et al*.)  ABR is not a significant problem nationally (disagree/strongly disagree) (Chuenchom *et al*.)  Students felt that ABR is a national problem (yes) (Haque *et al*. )  ABR is not a significant problem nationally (disagree/strongly disagree) (Yang *et al*.)  ABR is a national problem (yes) (Dyar Europe)  Do you think that ABR is a national problem? (yes) (Dyar France)  ABR is not a significant problem nationally (disagree/strongly disagree) (Abbo *et al*.)  ABR is an important and serious public health issue in our country (agree) (Khan *et al*.)  ABR is a problem in Democratic Republic of Congo (agree/strongly agree) (Thriemer *et al*.)  ABR has become a problem in China (agree) (Huang *et al*.) |  |  |  |  |  |  | 84% | | 87% | | 99% | | 84.6% | | 83% | | 91.6% | |  | |  | | 92% | | 94% | | 98% | | 88.7% | | 92.9% | | 82.6%  (5^th^ y)  67.2%  (1^st^ y) | |  | |  | |  |
| **TH,** *perception of antibiotic resistance as a problem at their teaching hospital:*  ABR is a serious health issue in our hospitals (somewhat agree/strongly agree) (Tayyab *et al*.)  ABR is a significant problem at the hospitals where I have rotated (agree/strongly agree) (Wasserman *et al*.)  Students thought that ABR was a problem in their own hospital (Hoque *et al*.)  ABR is not a significant problem in our hospital (disagree/strongly disagree) (Chuenchom *et al*.)  Students felt that ABR is a problem in their teaching hospital (yes) (Haque *et al*.)  ABR is not a significant problem at the hospitals where I have rotated (disagree/strong disagree) (Yang *et al*.)  ABR is a problem in my own hospital (yes) (Dyar Europe)  Do you think that ABR is a problem in your hospital? (yes) (Dyar *et al*. France)  ABR is not a significant problem at the hospitals where I have rotated (disagree/strongly disagree) (Abbo *et al*.)  ABR was an important and a serious public health issue in our hospital (agree) (Khan *et al*.) |  |  |  |  |  |  | 61% | | 61% | | 59% | | 75.4% | | 63% | | 85.0% | |  | |  | | 79% | | 69% | | 97% | | 68% | |  | |  | |  | |  | |  |
| **FC,** *perception of ABR as a problem in their future career:*  ABR will be a greater clinical problem later in my professional career than it is today (agree/strongly agree) (Rusic *et al*.)  Students believed that ABR would become a possible clinical problem during their career (yes)(Haque *et al*.)  Students believed that ABR would become a greater clinical problem during their career (Dyar Europe *et al*.)  Do you think that ABR will be a greater clinical problem later in your medical career than it is today? (highly likely/likely) (Dyar France *et al*.)  ABR is a problem in my practice (agree/strongly agree) (Thriemer *et al*.) |  | 84.6% |  |  |  |  |  | |  | |  | |  | | 42% | |  | |  | |  | | 98% | | 96% | |  | |  | | 67.4% | |  | |  | |  | |  |
| **ABOUP**, perception of antibiotics overused |  |  |  |  |  |  |  | |  | |  | |  | |  | |  | |  | |  | |  | |  | |  | |  | |  | |  | |  | |  | |  |
| **OUG,** *general overused:*  Antimicrobials are overused (agree/strongly agree) (Rusic *et al*.)  Is there abuse in antimicrobials at present (agree) (Sharma *et al*.)  There is abuse in antibiotics at present (agree) (Huang *et al*.))  Antibiotics are overused in general in outpatient medicine (strongly agree/somewhat agree) (Minen *et al*.) |  | 82.1% |  |  |  |  |  | |  | |  | |  | |  | |  | | 99.2% | |  | |  | |  | |  | |  | |  | | 91.7%  (5^th^ y)  87.2%  (1^st^ y) | | 79.5% | |  | |  |
| **OUN,** *overused nationally:*  AB are overused in South Africa (agree/strongly agree) (Wasserman *et al*.)  Antimicrobials are overused nationally (agree/strongly agree) (Chuenchom *et al*.)  Antimicrobials are overused nationally in healthcare (agree/strongly agree) (Yang *et al*.)  Antimicrobials are overused nationally (Abbo *et al*.) |  |  |  |  |  |  |  | | 92% | |  | | 98.2% | |  | | 84.8% | |  | |  | |  | |  | | 94% | |  | |  | |  | |  | |  | |  |
| **OUTH,** *overused at teaching hospitals:*  Antibiotics are overused at the hospitals where I have rotated (agree/strongly agree) (Wasserman *et al*.)  Antimicrobial are overused in our hospitals (agree/strongly agree) (Chuenchom *et al*.)  Antimicrobial are overused at the hospitals where I have rotated (agree/strongly agree) (Yang *et al*.)  Antimicrobial are overused at the hospitals where I have rotated (agree/strongly agree) (Abbo *et al*.)  Antibiotics are overused at hospitals I have worked in (strongly agree/somewhat agree) (Minen *et al*.) |  |  |  |  |  |  |  | | 63% | |  | | 94.3% | |  | | 39.4% | |  | |  | |  | |  | | 65% | |  | |  | |  | | 36.5% | |  | |  |
| **PCR**, perception of contributors to resistance | | | | | | | | | | | | | | | | | | | | | | | | | | | | | | | | | | | | | | |  |
| I**NH,** *inherent in the use of antibiotics:*  Appropriate use of antimicrobials can cause ABR (agree/strongly agree) (Rusic *et al*.)  Mutational and evolutionary changes in the microorganism (important) (Padmanabha *et al*.)  Appropriate use of antimicrobials can cause ABR (agree/strongly agree) (Chuenchom *et al*.)  Appropriate use of antimicrobials can cause ABR (agree/strongly agree) (Yang *et al*.)  Bacteria can become resistant to antibiotics (agree) (Harakeh *et al*.)  Appropriate use of antimicrobials can cause ABR (agree/strongly agree) (Abbo *et al*.) |  | 47.4% |  |  | 59.0% |  |  | |  | |  | | 85.3% | |  | | 24.6% | |  | | 95.0% | |  | |  | | 70% | |  | |  | |  | |  | |  | |  |
| **TMP,** *too many antibiotic prescriptions*:  Spread of resistant microorganisms in the presence of antimicrobials is facilitated by irrational use of drugs (Dutt *et al*.)  Too many antibiotic prescriptions (very important/moderately important) (Hoque *et al*.)  Too many antibiotic prescriptions (very important/moderately important) (Haque *et al*.)  Antimicrobial abuse is the leading cause of AR (agree) (Sharma *et al*.)  Too many antibiotic prescriptions (very important/moderately important) (Dyar *et al*. Europe)  Too many antibiotic prescriptions (very important/moderately important) (Dyar *et al*. France)  Lack of restrictions on antibiotic usage (important) (Khan *et al*.)  Too much prescription and consumption (yes) (Thriemer *et al*.)  Abuse of antibiotics has become the main cause leading to bacterial resistance (agree) (Huang *et al*.) |  |  |  | 53.9% |  |  |  | |  | | 67.3% | |  | | 88% | |  | | 96.7% | |  | | 95% | | 98% | |  | | 78.3% | | 69.0% | | 81.4%  (5^th^ y)  72.9%  (1^st^ y) | |  | |  | |  |
| **TMBS,** *too many broad-spectrum antibiotics used:*  Prescribing broad-spectrum antimicrobials when equally effective narrower-spectrum antimicrobials are available increases ABR (agree/strongly agree) (Rusic *et al*.)  Use of AB with a broader than necessary spectrum (important) (Padmanabha *et al*.)  Using a broad-spectrum antibiotic unnecessarily can increase resistance (agree/strongly agree) (Wasserman *et al*.**)**  Prescribing broad-spectrum antimicrobials increases ABR (agree/strongly agree) (Chuenchom *et al*.)  Too many broad-spectrum antibiotic used (very important/moderately important) (Hoque *et al*.)  Too many broad-spectrum antibiotics used (very important/moderately important) (Haque *et al*.)  Prescribing broad-spectrum antimicrobials when equally effective, narrower-spectrum antimicrobials are available increases ABR (agree/strongly agree) (Yang *et al*.)  Too many broad-spectrum antibiotics used (very important/moderately important) (Dyar *et al*. Europe)  Too many broad-spectrum antibiotics used (very important/moderately important) (Dyar *et al*. France)  Prescribing broad-spectrum antimicrobials when equally effective narrower-spectrum antimicrobials are available increases ABR (agree/strongly agree) (Abbo *et al*.)  Use of antibiotics with a broader than necessary spectrum (important) (Khan) |  | 83.3% |  |  | 36.7% |  |  | | 88% | | 66.4% | | 99.1% | | 86% | | 32.5% | |  | |  | | 96% | | 92% | | 95% | | 69% | |  | |  | |  | |  | |  |
| **TLT,** *too long treatment:*  Use of antibiotics for longer than standard duration (important)(Padmanabha *et al*.)  Using antibiotic treatment for a longer duration than what is indicated (some impact/great impact) (Weier *et al*.)  Too long durations of antibiotic treatment (very important/moderately important)(Hoque *et al*.)  Too long durations of antibiotic treatment (very important/moderately important)(Haque *et al*.)  Prolonged prophylactic therapy and prolonged empirical antimicrobial treatment without clear evidence of infection contribute to promoting ABR (Sharma *et al*.)  Too long durations of antibiotic treatment (very important/moderately important) (Dyar Europe *et al*.)  Too long durations of antibiotic treatment (very important/moderately important) (Dyar France *et al*.)  Use of antibiotic for longer than standard duration (important) (Khan *et al*.) |  |  |  |  | 47.5% | 75.2% |  | |  | | 54.2% | |  | | 72% | |  | | 100% | |  | | 55% | | 60% | |  | | 49.5% | |  | |  | |  | |  | |  |
| **TLD,** *too low dosage or treatment not completed:*  Use of antibiotic for shorter than standard duration (very important/moderately important) (Rusic *et al*.)  Use of antibiotic for shorter than standard duration (important) (Padmanabha *et al*.)  Patient non-compliance with antibiotic treatment (some impact/great impact) (Weier *et al*.)  Dosing of antibiotics are too low (very important/moderately important) (Hoque *et al*.)  Dosing of antibiotics are too low (very important/moderately important) (Haque *et al*.)  Dosing of antibiotics are too low (very important/moderately important) (Dyar *et al*. Europe)  Dosing of antibiotics are too low (very important/moderately important) (Dyar *et al*. France)  Use of antibiotics for shorter than standard duration (important) (Khan)  Too low dosage (yes) (Thriemer *et al*.)  Treatment not completed (yes) (Thriemer *et al*.) |  | 89.7% |  |  | 38.9% | 91.9% |  | |  | | 54.2% | |  | | 56% | |  | |  | |  | | 68% | | 63% | |  | | 58% | | 82.1%  89.7% | |  | |  | |  | |  |
| **IUAB,** *Inappropriate use of antibiotics:*  Inappropriate use of antimicrobials causes ABR (agree/strongly agree) (Rusic *et al*.)  Better use of antimicrobials will reduce problems with ABR (agree/strongly agree) (Rusic *et al*.)  Use of antibiotics for self-limited bacterial infections (very important/ moderately important) (Rusic *et al*.)  Spread of resistant microorganisms in the presence of antimicrobials is facilitated by misuse of drugs (Dutt)  Indiscriminate and injudicious use of antibiotics can lead to emergence of bacterial resistance (agree) (Dutt *et al*.)  Use of antibiotics for self-limited non-bacterial infections (important) (Padmanabha *et al*.)  Use of antibiotics for self-limited bacterial infections (important) (Padmanabha *et al*.)  Using the wrong antibiotics for the situation (some impact/great impact) (Weier *et al*.)  Prescribing antibiotics when the situation doesn’t warrant their use (some impact/great impact) (Weier *et al*.)  Inappropriate use of antibiotics causes ABR (agree/strongly agree) (Wasserman *et al*.)  Inappropriate use of antimicrobials can cause ABR (agree/strongly agree) (Yang *et al*.)  Use of antibiotics for viral infections contributes to promoting ABR (Sharma)  Inappropriate use of antimicrobials causes ABR (agree/strongly agree) (Abbo *et al*.)  Use of antibiotics for self-limited non bacterial infections (important) (Khan *et al*.)  Use of antibiotics for self-limited bacterial infections (important) (Khan *et al*.)  Antibiotics not adapted (yes) (Thriemer *et al*.) |  | 80.8%  88.5%  87.2% |  | 42.1%  78.9% | 35.3%  49.6% | 95.3%  96.6% |  | | 98% | |  | |  | |  | | 92.3% | | 100% | |  | |  | |  | | 97% | | 56.7%  56.7% | | 83.2% | |  | |  | |  | |  |
| **LSU,** *excessive use in livestock:*  Excessive use of antibiotics in livestock (very important/moderately important) (Rusic *et al*.)  Do you think that the overuse of antimicrobials in food production is also contributing to increased drug resistance? (yes) (Dutt *et al*.)  Excessive antibiotic use in livestock (animals reared for food) (important) (Padmanabha *et al*.)  Excessive use of antibiotic in livestock (very important/moderately important) (Hoque *et al*.)  Excessive use of antibiotic in livestock (very important/moderately important) (Haque *et al*.)  Excessive use of antibiotic in livestock (very important/moderately important) (Dyar *et al*. Europe)  Excessive use of antibiotic in livestock (very important/moderately important) (Dyar *et al*. France)  Excessive antibiotic use in livestock (animals reared for food) (important) (Khan *et al*.) |  | 83.3% |  | 65.8% | 48.9% |  |  | |  | | 65.5% | |  | | 75% | |  | |  | |  | | 76% | | 75% | |  | | 54.6% | |  | |  | |  | |  | |  |
| **PHH,** *poor hand hygiene:*  Poor infection control measures (important) (Padmanabha *et al*.)  Lack of hand hygiene by healthcare workers causes the spread of resistance (agree/strongly agree) (Wasserman *et al*.)  Poor hand hygiene (very important/moderately important) (Hoque *et al*.)  Poor adherence in hand hygiene practices can cause spread of ABR among patients (agree/strongly agree) (Chuenchom *et al*.)  Poor hand hygiene (very important/moderately important) (Haque *et al*.)  Poor hand hygiene (very important/moderately important) (Dyar *et al*. Europe)  Poor hand hygiene (very important/moderately important) (Dyar *et al*. France) |  |  |  |  | 33.1% |  |  | | 62% | | 60.7% | | 99.6% | | 49% | |  | |  | |  | | 42% | | 55% | |  | |  | |  | |  | |  | |  | |  |
| **PICM,** *poor infection control measures:*  Not removing the focus of infection (e.g., catheter) (very important/ moderately important) (Rusic *et al*.)  Not removing the focus of infection (very important/moderately important) (Hoque *et al*.)  Not removing the focus of infection (e.g., medical devices or catheters) (very important/moderately important) (Haque *et al*.)  Poor infection-control practices by healthcare professionals cause spread of ABR (agree/strongly agree) (Yang *et al*.)  Not removing the focus of infection (e.g., medical devices or catheters) (very important/moderately important) (Dyar *et al*. Europe)  Not removing the focus of infection (e.g., medical devices or catheters) (very important/moderately important) (Dyar *et al*. France)  Poor infection control practices by healthcare professionals cause spread of ABR (agree/strongly agree) (Abbo *et al*.)  Poor infection control measures (important) (Khan *et al*.)  In-hospital transmission (yes) (Thriemer *et al*.) |  | 88.5% |  |  |  |  |  | |  | | 81.3% | |  | | 78% | | 70.9% | |  | |  | | 67% | | 80% | | 83% | | 39.0% | | 64.1% | |  | |  | |  | |  |
| **ABRE,** attitude for integrating more training or education about antimicrobials and resistance | | | | | | | | | | | | | | | | | | | | | | | | | | | | | | | | | | | | | | |  |
| I feel I need more education on both general antibiotic treatment and prudent antibiotic use. (Sanchez-Fabra *et al*.)  I would like more education on the appropriate use of antimicrobials (agree/strongly agree) (Rusic *et al*.)  I would like more education on ABR (agree/strongly agree) (Rusic *et al*.)  I would appreciate more education on the appropriate use of antibiotics (agree/strongly agree) (Wasserman *et al*.)  I would appreciate more education on ABR in general (agree/strongly agree) (Wasserman *et al*.)  I would like more education on how to use antimicrobials appropriately (Chuenchom *et al*.) (agree/strongly agree)  I would like more education on ABR (agree/strongly agree) (Chuenchom *et al*.)  I would like more education on hospital infection control (agree/strongly agree) (Chuenchom *et al*.)  Study participants stated that they would like more training in the antibiotic selection (Haque *et al*.)  I would like more education on ABR (agree/strongly agree) (Yang *et al*.)  I would like more education on the appropriate use of antimicrobials (agree/strongly agree) (Yang *et al*.)  Medical students had a positive attitude for integrating antimicrobial pharmacology teaching with the clinical subject of medicine (Sharma *et al*.)  Students stated that they would like more training on antibiotic selection (Dyar *et al*. Europe)  Students stated that they would like more training on antibiotic selection (Dyar *et al*. France)  I would like more education on the appropriate use of antimicrobials (Abbo *et al*.)  I would like more education on ABR (Abbo *et al*.)  Necessary to get more education about antibiotics (agree) (Huang *et al*.)  Need to establish course on “rational use of antibiotics at university” (agree) (Huang *et al*.)  I would like more ongoing education on antibiotics (strongly agree/somewhat agree) (Minen *et al*.) | 40.6% | 83.3%  74.4% |  |  |  |  |  | | 90%  95% | |  | | 99.8%  99.6%  98.9% | | 88% | | 90.4%  85.9% | | 98% | |  | | 67%-  92% | | 79% | | 90%  79% | |  | |  | | 87.0%  74.5%  (5^th^ y)  89.3%  61.1  (1^st^ y) | | 65.8% | |  | |  |
| **PPAB,** medical students’ perceptions of preparedness in antimicrobials | | | | | | | | | | | | | | | | | | | | | | | | | | | | | | | | | | | | | | |  |
| Do you feel you have received sufficient teaching at medical school in antibiotic use for your future practice as a junior doctor?    This section asks how well you feel your studies at medical school have prepared you for your practice as a junior doctor on the following topics of prudent antibiotic use (prepared). (Sanchez-Fabra *et al*.)  -To recognise the clinical signs of infection  -To assess the clinical severity of infection (e.g., using criteria, such as septic shock criteria)  -To use point‐of‐care tests (e.g., urine dipstick, rapid diagnostic tests for streptococcal pharyngitis)  -To interpret biochemical markers of inflammation (e.g., CRP)  -To decide when it is important to take microbiological samples before starting antibiotic therapy  -To interpret basic microbiological investigations (e.g., blood cultures, antibiotic susceptibility reporting)  -To select initial empirical therapy based on the most likely pathogen(s) and antibiotic resistance patterns, without using guidelines  -To decide the urgency of antibiotic administration in  different situations (e.g., <1 hr for severe sepsis, non‐urgent for  chronic bone infections)  -To prescribe antibiotic therapy according to national/local guidelines  -To assess antibiotic allergies (e.g., differentiating between anaphylaxis and hypersensitivity)  -To identify indications for combination antibiotic therapy  -To decide the shortest possible adequate duration of antibiotic  therapy for a specific infection  -To review the need to continue or change antibiotic therapy after  48‐72 hours, based on clinical evolution and laboratory results  -To decide when to switch from intravenous (IV) to oral antibiotic therapy  -Discuss with the patient the use of antibiotics when I think they are not necessary and he/she asks for them  -To use knowledge of the common mechanisms of antibiotic resistance in pathogens  -To use knowledge of the epidemiology of bacterial resistance,  including local/regional variations  -To practise effective Infection control and hygiene (to prevent  spread of bacteria)  -To use knowledge of the negative consequences of antibiotic use  (bacterial resistance, toxic/adverse effects, cost, *Clostridium*  *difficile* infections) | 24.3%  94.8%  81.2%  66.2%  86.6%  78.2%  78.1%  40.4%  48.2%  43.6%  46.2%  38.7%  34.7%  55.5%  50.3%  79.8%  63%  44%  84.4%  84.1% |  |  |  |  |  |  | |  | |  | |  | |  | |  | |  | |  | |  | |  | |  | |  | |  | |  | |  | |  | |  |
| Students who feel their education has prepared them well/very well for practice related to ABR (Rusic *et al*.)  -Understand basics of ABR  -How to interpret antibiograms  -How to find reliable sources of information to treat infections  -How to handle patient who demands antimicrobials  -To prescribe the correct spectrum of different antimicrobials therapies  -How to select the best antimicrobial for a specific infection  -To know when to start antimicrobial therapy |  | 69.2%  74.4%  61.5%  53.8%  50.0%  48.7%  44.9% |  |  |  |  |  | |  | |  | |  | |  | |  | |  | |  | |  | |  | |  | |  | |  | |  | |  | |  | |  |
| Students were asked to report the sufficiency of education they received at university in ID and antibiotic prescribing (sufficient/more than sufficient) |  |  |  |  |  | 67% |  | |  | |  | |  | |  | |  | |  | |  | |  | |  | |  | |  | |  | |  | |  | |  | |  |
| Medical student´s perceptions of preparedness in antimicrobial stewardship (good/very good)  -Making accurate diagnosis of infection  -Knowing when to start antibiotics  -Choosing the correct antibiotic  -Knowledge of dosing and duration  -How to de-escalate to narrower spectrum  -How and when to transition from intravenous to oral  -How to interpret antibiograms  -Understanding spectrums of activity  -Understanding basic mechanisms of resistance |  |  |  |  |  |  |  | | 87%  66%  46%  32%  26%  46%  13%  46%  57% | |  | |  | |  | |  | |  | |  | |  | |  | |  | |  | |  | |  | |  | |  | |  |
| -I have learned enough about appropriate use of antimicrobials (good perception)  -I can access reliable sources of knowledge on antimicrobials  -I know which patient needs to be treated with antimicrobials  -I can prescribe the appropriate antimicrobial to patients  -I am aware of appropriate antimicrobial use in routine patient care  -I know how to prevent and control spread of ABR  -I know when I have to wear a surgical mask for routine patient care |  |  |  |  |  |  |  | |  | |  | | 87.2%  97.2%  99.6%  98.0%  99.7%  96.9%  98.7% | |  | |  | |  | |  | |  | |  | |  | |  | |  | |  | |  | |  | |  |
| Medical students’ perceptions of their education regarding appropriate antimicrobial use and antimicrobial stewardship. (good/very good)  -Understand the basic mechanism of antimicrobial resistance  -Know when to start antimicrobial therapy  -Select an appropriate regimen  -Describe the correct spectrum of antimicrobial therapy for different antimicrobials (what is covered by each drug)  -Transition from intravenous to oral antimicrobials (intravenous to oral switch)  -Find reliable sources of information to treat infections  -Interpret antibiograms  -Streamline or de-escalate antimicrobial therapy  -Handle a patient who demands antimicrobial therapy that is not indicated |  |  |  |  |  |  |  | |  | |  | |  | |  | | 54.1%  40.0%  36.9%  35.2%  34.2%  34.0%  28.3%  28.2%  25.6% | |  | |  | |  | |  | |  | |  | |  | |  | |  | |  | |  |
| Percentage who feel their medical education has been good/very good:  -Find reliable sources of information to treat infections  -Basic mechanisms of ABR  -Handle patients demanding unnecessary antibiotics  -When to start antimicrobial therapy  -Select the best antimicrobial for a specific infection  -Spectrum of therapy for different antibiotic  -Interpret antibiograms  -Transition intravenous to oral antibiotic  -Streamline antibiotic therapy |  |  |  |  |  |  |  | |  | |  | |  | |  | |  | |  | |  | |  | |  | | 63%  61%  54%  54%  39%  34%  34%  32%  31% | |  | |  | |  | |  | |  | |  |
| **CABK,** confidence in antibiotic knowledge or antibiotic prescription | | | | | | | | | | | | | | | | | | | | | | | | | | | | | | | | | | | | | | |  |
| Confidence in knowledge in different clinical situations (somewhat confident/most confident)  -Accurately diagnosing community-acquired pneumonia  -Accurately interpreting pathology and microbiology results  -Identifying situations where antibiotic treatment is not necessary  -knowing the right regimen for the antibiotic treatment for a specific indication such as pneumonia or an exacerbation of chronic obstructive pulmonary disease  -Knowing when antibiotic treatment needs to be adjusted, stopped, or other treatment needs to be used  -Knowing the right duration for antibiotic treatment for a specific indication such as pneumonia or an exacerbation of chronic obstructive pulmonary disease. |  |  |  |  |  | 89.7%  89.0%  67.7%  55.5%  54.2%  38.8% |  | |  | |  | |  | |  | |  | |  | |  | |  | |  | |  | |  | |  | |  | |  | |  | |  |
| Levels of confidence in antibiotic prescribing (confident/very confident):  -Making an accurate diagnosis of infection/sepsis  -Deciding not to prescribe antimicrobials if the patient has a fever but no severe criteria, and if you are not sure about your diagnosis  -Choosing the correct antimicrobial  -Choosing the correct dose and interval of administration  -Using a combination therapy if appropriate  -Choosing between IV and oral administration  -Interpreting microbiological results  -Planning to streamline/stop the antimicrobial treatments, according to clinical evaluation and investigations  -Planning the duration of the MB treatment |  |  |  |  |  |  |  | |  | | 77.5%  64.5%  72.9%  75.7%  71.9%  76.6%  75.7%  72.8%  70.1% | |  | |  | |  | |  | |  | |  | |  | |  | |  | |  | |  | |  | |  | |  |
| -I feel anxious when I have to prescribe antimicrobial agents (negative). |  |  |  |  |  |  |  | |  | |  | | 71.4% | |  | |  | |  | |  | |  | |  | |  | |  | |  | |  | |  | |  | |  |
| I feel confident or very confident  -Making an accurate diagnosis of infection/sepsis  -Interpreting microbiological results  -Choosing the correct antibiotic  -Choosing the correct dose and interval of administration  -Using a combination therapy if appropriate  -Choosing between intravenous and oral administration  -Deciding not to prescribe an antibiotic if the patient has fever, but no severity criteria, and if you are not sure about your diagnosis  -Planning to streamline/stop the antibiotic treatment according to the clinical evaluation and investigations  -Planning the duration of the antibiotic treatment |  |  |  |  |  |  |  | |  | |  | |  | | 80.3%  58.3%  66.9%  48.5%  53.6%  73.9%  64.8%  62.0%  59.2% | |  | |  | |  | |  | |  | |  | |  | |  | |  | |  | |  | |  |
| Are you competent enough to select the best antimicrobial for the following scenarios?  -Community-acquired pneumonia  -Complicated UTI.  -Multiresistant *Escherichia coli* infection  -MRSA infection  -Multi-drug-resistant *Acinetobacter*  -Multi-drug-resistant *Pseudomonas aeruginosa*.  -Drug-resistant *Salmonella Typhi*  -Drug-resistant tubercular infection (multi-drug-resistant and extensively drug-resistant TB)  -Vancomycin resistant *Enterococc*i  Are your competent enough to identify the scenarios where antimicrobials are not required (Sharma) |  |  |  |  |  |  |  | |  | |  | |  | |  | |  | | 52.5%  70.8%  47.5%  69.2%  7.5%  65.8%  81.6%  53.3%  70.8%  93.3% | |  | |  | |  | |  | |  | |  | |  | |  | |  | |  |
| I feel confident or very confident:  -Using a combination therapy if appropriate  -Choosing between intravenous and oral administration  -Deciding not to prescribe an antibiotic if the patient has fever, but no severity criteria, and if you are not sure about your diagnosis  -Planning to streamline/stop the antibiotic treatment according to the clinical evaluation and investigations  -Planning the duration of the antibiotic treatment  -Choosing the correct dose and interval of administration  -Choosing the correct antibiotic  -Making an accurate diagnosis of infection/sepsis  -Interpreting microbiological results |  |  |  |  |  |  |  | |  | |  | |  | |  | |  | |  | |  | | 40%  74%  48%  61%  59%  46%  73%  92%  83% | |  | |  | |  | |  | |  | |  | |  | |  |
| I feel confident or very confident:  -Planning the duration  -Planning to streamline/stop the treatment  -Interpreting microbiological results  -Choosing between intravenous and oral administration  -Using a combination therapy if appropriate  -Choosing the correct dose and interval of administration  -Choosing the correct antibiotic  -Not prescribing an antibiotic in case of fever with diagnostic uncertainly  -Making an accurate diagnosis of infection/sepsis |  |  |  |  |  |  |  | |  | |  | |  | |  | |  | |  | |  | |  | | 50%  62%  83%  78%  65%  33%  70%  65%  97% | |  | |  | |  | |  | |  | |  | |  |
| Medical students stated that they felt very confident or confident about their knowledge on antibiotics |  |  |  |  |  |  |  | |  | |  | |  | |  | |  | |  | |  | |  | |  | |  | |  | | 85.7% | |  | |  | |  | |  |
| **RSP**, responsibility | | | | | | | | | | | | | | | | | | | | | | | | | | | | | | | | | | | | | | |  |
| **OPR**, *own professional responsibility****:***  antimicrobials that I will prescribe will contribute to the problem of resistance (agree/strongly agree) (Rusic *et al*.)  Prescribing inappropriate or unnecessary antimicrobials is professionally unethical (agree/strongly agree) (Rusic *et al*.)  Prescribing inappropriate or unnecessary antibiotics is professionally unethical (Haque *et al*.)  Students felt that the antibiotics they would prescribe would contribute to ABR (Dyar *et al*. Europe)  Do you believe that the antibiotics you will prescribe as a physician will contribute to the problem of resistance? (highly likely/likely) (Dyar *et al*. France)  Whenever I take an antibiotic, I contribute to the development of ABR (agree) (Khan *et al*.) |  | 48.7%  79.5% |  |  |  |  |  | |  | |  | |  | | 87% | |  | |  | |  | | 66% | | 63% | |  | | 53.6% | |  | |  | |  | |  | |  |
| **ABDP**, *perception about development of new antibiotics****:***  New antibiotics are available to deal with the problem of resistance (agree/strongly agree) (Wasserman *et al*.)  Students thought that there would be six to ten new antibiotic classes in 2011-2020. (Hoque *et al*.)  New antimicrobials will be developed in the future to solve resistance. (Agree/strongly agree) (Chuenchom *et al*.)  Students thought that there would be six to ten new antibiotic classes in 2011-2020. (Haque *et al*.)  New antimicrobials will be developed in the future that will keep up with the problem of “resistance” (agree/strongly agree) (Yang)  New antimicrobials could be developed in future that will keep up with the problem of AR (agree) (Sharma *et al*.)  Students thought that more new antibiotic classes would become available in this decade as compared to 1980-2011 (Dyar *et al*. Europe)  Students believe that at least one new class of antibiotic would become available between 2011-2020. (Dyar *et al*. Europe)  Students thought that more new antibiotic classes would become available in this decade as compared to 1980-2011 (Dyar *et al*. France)  New antimicrobials will be developed in the future that will keep up with the problem of "resistance" (agree/strongly agree) (Abbo *et al*.) |  |  |  |  |  |  |  | | 13% | | 32.7% | | 99.1% | | 23% | | 76.9% | | 72.5% | |  | | 40%  89% | | 74% | | 20% | |  | |  | |  | |  | |  | |  |
| **Abbreviations:**  ABR: antibiotic resistance |  |  |  |  |  |  |  | |  | |  | |  | |  | |  | |  | |  | |  | |  | |  | |  | |  | |  | |  | |  | |  |
