# Supplementary material for: Does the problem begin at the beginning? Medical students’ knowledge and beliefs regarding antibiotics and resistance: a systematic review
Source: Antimicrob Resist Infect Control. 2020 Nov 3;9:172. doi: 10.1186/s13756-020-00837-z (PMC7607835; doi:10.1186/s13756-020-00837-z)
Supplement: Supplementary file 3 — Additional file 3. Antibiotic behaviour among medical students. [file 13756_2020_837_MOESM3_ESM.docx]

**Additional File 3.** Antibiotic behaviour among medical students

|  | Sanchez-Fabra *et al.* | Rusic *et al.* | Hu *et al.* | Dutt *et al.* | Padmanabha *et al.* | Weier *et al.* | Tayyab *et al.* | W asserman *et al.* | Hoque *et al.* | Chuenchom *et al.* | Haque *et al.* | Yang *et al.* | Sharma *et al.* | Harakeh *et al.* | Dyar *et al*.(Europe) | Dyar *et al* .(France) | Abbo *et al.* | Khan *et al*. | Thriemer *et al*. | Huang *et al*. | Minen *et al.* | Ibia *et al.* |
| --- | --- | --- | --- | --- | --- | --- | --- | --- | --- | --- | --- | --- | --- | --- | --- | --- | --- | --- | --- | --- | --- | --- |
|  | Sample size (n) | | | | | | | | | | | | | | | | | | | | | |
|  | 441 | 78 | 1819 | 76 | 139 | 191 | 223 | 289 | 107 | 455 | 142 | 611 | 120 | 1042 | 338 | 60 | 317 | 97 | 106 | 1236 | 304 | 989 |
| **SSM**, *student’s self-medication with antibiotics and* **SMRTI** *self-medication with antibiotics for respiratory tract infections* |  | | | | | | | | | | | | | | | | | | | | | |
| Antibiotic use behaviour for self-limiting illness symptoms among medical students in China in 2015    -Self-medication with antibiotics (general) **SSM**  -for cold **SMRTI**  -for fever  -for sore throat **SMRTI**  -for ear pain  -for headache  -for flu-like illness **SMRTI**  -for diarrhoea  -for abdominal pain |  |  |  |  |  |  |  |  |  |  |  |  |  |  |  |  |  |  |  |  |  |  |
|  |  |  | 27.0% |  |  |  |  |  |  |  |  |  |  |  |  |  |  |  |  |  |  |  |
|  |  |  | 30.7% |  |  |  |  |  |  |  |  |  |  |  |  |  |  |  |  |  |  |  |
|  |  |  | 33.3% |  |  |  |  |  |  |  |  |  |  |  |  |  |  |  |  |  |  |  |
|  |  |  | 34.6% |  |  |  |  |  |  |  |  |  |  |  |  |  |  |  |  |  |  |  |
|  |  |  | 55.6% |  |  |  |  |  |  |  |  |  |  |  |  |  |  |  |  |  |  |  |
|  |  |  | 25.5% |  |  |  |  |  |  |  |  |  |  |  |  |  |  |  |  |  |  |  |
|  |  |  | 55.6% |  |  |  |  |  |  |  |  |  |  |  |  |  |  |  |  |  |  |  |
|  |  |  | 32.0% |  |  |  |  |  |  |  |  |  |  |  |  |  |  |  |  |  |  |  |
|  |  |  | 16.0% |  |  |  |  |  |  |  |  |  |  |  |  |  |  |  |  |  |  |  |
| Have you ever self-medicated with antibiotics? (sometimes/always) **SSM**  If option above is marked yes, then for which of the following complaints did you use antibiotics?  -Sore throat **SMRTI**  -Fever  - Diarrhoea |  |  |  | 76.4% |  |  |  |  |  |  |  |  |  |  |  |  |  |  |  |  |  |  |
|  |  |  |  |  |  |  |  |  |  |  |  |  |  |  |  |  |  |  |  |  |  |  |
|  |  |  |  | 48.7% |  |  |  |  |  |  |  |  |  |  |  |  |  |  |  |  |  |  |
|  |  |  |  | 39.5% |  |  |  |  |  |  |  |  |  |  |  |  |  |  |  |  |  |  |
|  |  |  |  | 10.5% |  |  |  |  |  |  |  |  |  |  |  |  |  |  |  |  |  |  |
| Do you consult a doctor before starting an antibiotic? (sometimes/seldom/never) **SSM**  Do you prefer to take antibiotics when you have a cough and sore throat? (sometimes/ always/usually) **SMRTI** |  |  |  |  |  |  | 41% |  |  |  |  |  |  |  |  |  |  |  |  |  |  |  |
|  |  |  |  |  |  |  | 82% |  |  |  |  |  |  |  |  |  |  |  |  |  |  |  |
| Students had used antibiotics during the past year and these medicines were not obtained under a doctor's prescription **SSM**  Students had used antibiotics during the past year and were used for an RTI. **SMRTI** |  |  |  |  |  |  |  |  |  |  |  |  |  | 49% |  |  |  |  |  |  |  |  |
|  |  |  |  |  |  |  |  |  |  |  |  |  |  | 61.8% |  |  |  |  |  |  |  |  |
| Do you consult a doctor before starting antibiotics? (sometimes/seldom/never) **SSM**  Do you prefer to take an antibiotic when you have cough and sore throat? (sometimes/ always/usually) **SMRTI**  When I have a cold, I should take antibiotics to prevent getting a more serious illness **SMRTI** |  |  |  |  |  |  |  |  |  |  |  |  |  |  |  |  |  | 7.2% |  |  |  |  |
|  |  |  |  |  |  |  |  |  |  |  |  |  |  |  |  |  |  | 59.8% |  |  |  |  |
|  |  |  |  |  |  |  |  |  |  |  |  |  |  |  |  |  |  | 56% |  |  |  |  |
| Use antibiotics when having... (always, often)  -Fever (temperature lower than 38.5º)  -Common cold (always, often) **SMRTI**  -Acute bronchitis (always, often) **SMRTI**  -Coughing up yellow/green sputum (always, often) **SMRTI**  -Sore throat (always, often) **SMRTI**  -Cough with fever (always, often) **SMRTI**  -Congested nose with headache (always, often) **SMRTI**  -Coughing up white sputum (always, often) **SMRTI**  -Cough lasting 2 weeks or more **SMRTI**  -Ask doctors to prescribe antibiotics when you catch a common cold (yes) (Huang) **SMRTI** |  |  |  |  |  |  |  |  |  |  |  |  |  |  |  |  |  |  |  |  |  |  |
|  |  |  |  |  |  |  |  |  |  |  |  |  |  |  |  |  |  |  |  | 55.6%  (5^th^ y) |  |  |
|  |  |  |  |  |  |  |  |  |  |  |  |  |  |  |  |  |  |  |  | 39.7%  (1^st^ y)  14.5%  (5^th^ y)  6.4%  (1^st^ y) |  |  |
|  |  |  |  |  |  |  |  |  |  |  |  |  |  |  |  |  |  |  |  | 39.8%  (5^th^ y)  14.2%  (1^st^ y) |  |  |
|  |  |  |  |  |  |  |  |  |  |  |  |  |  |  |  |  |  |  |  | 56.4%  (5^th^ y)  14.9%  (1^st^ y) |  |  |
|  |  |  |  |  |  |  |  |  |  |  |  |  |  |  |  |  |  |  |  | 20.8%  (5^th^ y)  10.6%  (1^st^ y) |  |  |
|  |  |  |  |  |  |  |  |  |  |  |  |  |  |  |  |  |  |  |  | 28.6%  (5^th^ y)  12.1%  (1^st^ y) |  |  |
|  |  |  |  |  |  |  |  |  |  |  |  |  |  |  |  |  |  |  |  | 15.8%  (5^th^ y)  10.6%  (1^st^ y) |  |  |
|  |  |  |  |  |  |  |  |  |  |  |  |  |  |  |  |  |  |  |  | 17.8%  (5^th^ y)  6.4%  (1^st^ y) |  |  |
|  |  |  |  |  |  |  |  |  |  |  |  |  |  |  |  |  |  |  |  | 35.7%  (5^th^ y)  20.6%  (1^st^ y) |  |  |
|  |  |  |  |  |  |  |  |  |  |  |  |  |  |  |  |  |  |  |  | 29.8%  (5^th^ y)  8.6%  (1^st^ y) |  |  |
| **ICU**, *incorrect use of antibiotics* |  | | | | | | | | | | | | | | | | | | | | | |
| Do you give the leftover antibiotics to your friends/roommates if they get sick? (always/sometimes)  Do you complete the full course of treatment (not always) |  |  |  | 50%  28.9% |  |  |  |  |  |  |  |  |  |  |  |  |  |  |  |  |  |  |
| The doctor prescribes a course of antibiotics for you. After taking 2-3 doses you start feeling better:  -Do you stop taking further treatment? (always/usually/sometimes)  -Do you save remaining antibiotics for next time you get sick? (always/usually/sometimes)  -Do your discard the remaining medicine? (sometimes/never/seldom)  -Do you give leftover medicine to your friends if they get sick? (always/usually/sometimes)  -Do you complete the full course of treatment? (sometimes/seldom/never)  -Do you check the expiry date of antibiotics before using them? (sometimes/seldom/never) |  |  |  |  |  |  | 61%  59%  78%  62%  31%  14% |  |  |  |  |  |  |  |  |  |  |  |  |  |  |  |
| The doctor prescribes a course of antibiotics for you. After taking 2-3 doses you start feeling better:  -Do your stop taking the further treatment? (sometimes/always/ usually)  -Do you save the remaining antibiotics for the next time you get sick? (always/usually/ sometimes)  -Do you give the leftover antibiotics to your friends/roommates if they get sick? (sometimes/always/usually)  -Do you discard the remaining leftover medication? (sometimes/seldom/never)  -Do you complete the full course of treatment? (sometimes, seldom or never)  -Do you check the expiry date of the antibiotic before using it? (sometimes/seldom/never) |  |  |  |  |  |  |  |  |  |  |  |  |  |  |  |  |  |  |  |  |  |  |
|  |  |  |  |  |  |  |  |  |  |  |  |  |  |  |  |  |  | 44.3% |  |  |  |  |
|  |  |  |  |  |  |  |  |  |  |  |  |  |  |  |  |  |  | 40.2% |  |  |  |  |
|  |  |  |  |  |  |  |  |  |  |  |  |  |  |  |  |  |  | 53.6% |  |  |  |  |
|  |  |  |  |  |  |  |  |  |  |  |  |  |  |  |  |  |  | 69% |  |  |  |  |
|  |  |  |  |  |  |  |  |  |  |  |  |  |  |  |  |  |  | 25.4% |  |  |  |  |
|  |  |  |  |  |  |  |  |  |  |  |  |  |  |  |  |  |  | 13.4% |  |  |  |  |
